# Cardiac fibroblast heterogeneity in cardiac fibrosis implication for cell-type-specific treatment

**DOI:** 10.3389/fphys.2026.1740147

**Published:** 2026-03-26

**Authors:** Kai Gu, Dongyan Song, Jisheng Chen

**Affiliations:** Department of Cardiology, The First People’s Hospital of Lin’an District, Lin’an People’s Hospital Affiliated to Hangzhou Medical College, Hangzhou, Zhejiang, China

**Keywords:** cardiac fibrosis, fibroblast heterogeneity, immune-fibroblast crosstalk, single-cell transcriptomics, targeted therapy

## Abstract

Cardiac fibrosis is a central feature of heart disease, driven by excessive extracellular matrix (ECM) accumulation and associated with mechanical stiffening, electrical instability, and heart failure. While cardiac fibroblasts (CFs) were historically viewed as a uniform ECM-producing population, lineage tracing and single-cell/spatial transcriptomics reveal substantial fibroblast heterogeneity in healthy myocardium and distinct activation trajectories in disease. This review summarizes fibroblast states in the uninjured heart and the dynamic emergence of specialized fibroblast subpopulations after ischemic injury (myocardial infarction) and during chronic pressure overload. We highlight immune-fibroblast crosstalk as key determinants of fibroblast fate, and discuss how these insights enable precision anti-fibrotic strategies that target pathogenic subtypes while preserving adaptive repair.

## 1 Introduction

Cardiac fibrosis, fundamentally characterized by the excessive accumulation of ECM proteins within the myocardial interstitium and perivascular spaces, stands as a common pathological denominator in nearly all forms of heart disease (Moore-Morris et al., 2015; Kong et al., 2014). The accumulation of ECM increases myocardial stiffness, impairing both diastolic filling and potentially systolic contraction (Snider et al., 2009). Furthermore, fibrosis disrupts the coordinated electrical activation of the heart by creating conduction barriers and altering cell-cell coupling, thereby establishing a substrate for potentially lethal arrhythmias (Frangogiannis, 2021). As a key component of adverse cardiac remodeling, fibrosis significantly contributes to the progression of heart failure (HF), impacting both patients with preserved (HFpEF) and reduced ejection fraction (HFrEF) (Ghazal et al., 2025). Given its association with morbidity and mortality, fibrosis remains a major unmet therapeutic target (Hinderer and Schenke-Layland, 2019).

Cardiac fibroblasts (CFs) are a major non-myocyte constituent of the mammalian heart and are often estimated to comprise ∼10%–30% of human cardiac cells, although proportions vary by species and methodology (Harrington and Moore-Morris, 2024; Pinto et al., 2016). Early studies emphasized ECM production (Moore-Morris et al., 2015), but subsequent work established CFs as mechanosensitive and paracrine signaling cells capable of phenotypic plasticity (Lajiness and Conway, 2012; Mouton et al., 2019). Most recently, lineage tracing and single-cell transcriptomics have refocused the field on fibroblast heterogeneity as a core feature of both homeostasis and pathological remodeling, positioning CF subpopulations as key orchestrators of the cardiac microenvironment (Umbarkar et al., 2021).

Historically, CFs were often identified using immunostaining for biomarkers, including vimentin, Thy1 (also known as CD90), and fibroblast specific protein-1 (FSP1/S100A4), because these were readily detectable on spindle-shaped interstitial cells and seemed to label mesenchymal or stromal compartments before robust genetic tools were widespread. However, these markers failed as definitive fibroblast identifiers mainly because they are not cell-type specific and because fibroblasts themselves are heterogeneous and state-dependent, so any single marker both mislabels other lineages and misses fibroblast subsets. Cardiac remodeling analyses showed that FSP1 is predominantly expressed by hematopoietic and endothelial cells in the injured heart, meaning “FSP1^+^ fibroblasts” often were not fibroblasts at all (Kong et al., 2013). Vimentin is highly sensitive for mesenchymal cells but is widely expressed by endothelium, smooth muscle cells, and pericytes, making it unreliable for fibroblast attribution in remodeling tissues (Zeisberg and Kalluri, 2010). Thy1 is similarly non-specific, with expression reported in multiple stromal and non-stromal compartments (including immune, endothelial and perivascular cells), and it labels only subsets of fibroblasts. Therefore, “Thy1^+^ fibroblast” can both overcall and undercall fibroblasts depending on context (Ivey and Tallquist, 2016). These limitations drove the field toward combinatorial marker panels with exclusion gating, genetic lineage tracing, and single-cell/spatial transcriptomics to define fibroblasts by integrated identity rather than single-marker staining (Tallquist and Molkentin, 2017).

In this review, we aim to provide a comprehensive overview of the current understanding of cardiac fibroblast heterogeneity, exploring its origins, phenotypic diversity, functional implications in cardiac fibrosis, and the potential therapeutic avenues opened by this evolving paradigm.

## 2 Cardiac fibroblasts diversity in cardiac fibrosis

### 2.1 Fibroblast subtypes in healthy heart

**FIGURE 1:**
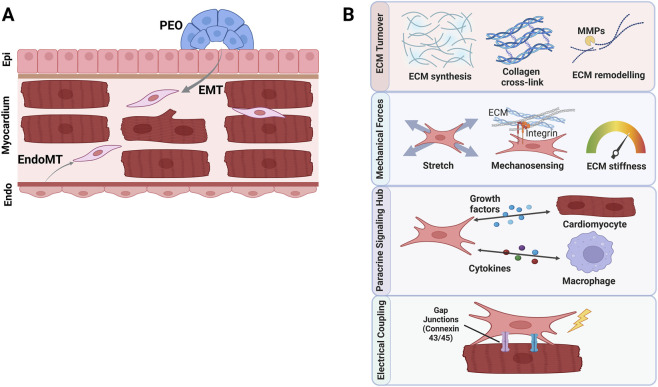
Developmental origins and physiological functions of cardiac fibroblasts. **(A)** Schematic representation of fibroblast origins. The majority of resident ventricular fibroblasts originate from the pro-epicardial organ (PEO) via epithelial-to-mesenchymal transition (EMT). A secondary population derives from endocardial cells through endothelial-to-mesenchymal transition (EndoMT). **(B)** Overview of the primary homeostatic functions of cardiac fibroblasts (CFs) in the healthy heart: ECM synthesis and turnover; Mechanosensing and mechanotransduction; Paracrine signaling hub interacting with immune cells (macrophages) and cardiomyocytes; and Electrical coupling with cardiomyocytes via gap junctions to modulate conduction. Epi: epicardium; Endo: endocardium.

In the healthy heart, fibroblast subtypes vary by developmental origin, anatomical location, and molecular phenotype (Figure 1A). The primary origin of ventricular CFs during embryonic development is the pro-epicardial organ (PEO). Cells from the PEO undergo epithelial-to-mesenchymal transition (EMT), migrate into the developing myocardium, and differentiate into fibroblasts (Lajiness and Conway, 2012). There are also proposed contributions from other sources such as endocardial-derived cells through endothelial-to-mesenchymal transition (EndoMT) and bone marrow-derived progenitors (Lajiness and Conway, 2012). However, genetic lineage tracing studies have demonstrated that the fibroblasts contributing to pathological fibrosis largely originate from resident fibroblast populations that expand and become activated in response to injury (Tallquist and Molkentin, 2017).

Recent single-cell RNA sequencing (scRNA-seq) and spatial approaches show that fibroblasts in the uninjured adult heart occupy multiple transcriptional states and localize to distinct niches (interstitial vs. perivascular) and chambers (Kanemaru et al., 2023). Using scRNA-seq, Patrick et al. integrated healthy heart datasets and defined five principal fibroblast subtypes in mouse (Patrick et al., 2024). These subtypes are characterized by differential expression of platelet-derived growth factor receptor alpha (PDGFRα, a pan-fibroblast marker) and various stem cell and extracellular matrix genes. Key subpopulations and their biomarkers identified include: (1) Stem cells antigen-1 (Sca1, encoded by the Ly6a gene, which stands for lymphocyte antigen-6 complex, locus A) high progenitor-like fibroblasts (F-SH) that co-express PDGFRα with high Sca1 plus stem/progenitor markers such as peptidase inhibitor 16 (Pi16); (2) Sca1 low quiescent matrix fibroblasts (F-SL), still PDGFRα positive but low for Sca1 and selectively enriched for C-X-C motif chemokine ligand 14 (Cxcl14) and hydroxysteroid 11-beta dehydrogenase 1 (Hsd11b1), reflecting a mature, ECM-maintaining state; (3) Wnt-modulating fibroblasts (F-WntX), a distinct subset with high expression of secreted Wnt inhibitory factor 1 (Wif1) and Secreted frizzled related protein 2 (Sfrp2), implying a homeostatic brake on profibrotic Wnt signalling; (4) Transitional fibroblasts (F-Trans) that transcriptionally bridge F-SL and F-WntX and are marked by upregulation of fibrinogen-like protein 2 (Fgl2) and insulin like growth factor binding protein 3 (Igfbp3); (5) Fibroblast-activated (F-Act) cells, moderately abundant in healthy hearts and increasing after myocardial infarction, are characterized by the graded expression of canonical activation markers like periostin (POSTN) and collagen type VIII alpha 1 (Col8a1) while showing diminished levels of stem-related markers such as Ly6a.

Human heart likewise contains diversified fibroblast programs. A comprehensive cell atlas of the adult human heart (Litvinukova et al., 2020) resolves seven fibroblast states (FB) that all share the pan-fibroblast markers Decorin (DCN) and PDGFRα. Ventricular-enriched FB1 and atrial-enriched FB2 form a basal, chamber-specific programme and are thought to explain the stronger profibrotic tendency noted for atrial fibroblasts. FB3, relatively depleted in the left ventricle, suppresses extracellular-matrix genes while up-regulating cytokine-receptor transcripts oncostatin M receptor (OSMR) and interleukin 6 signal transducer (IL6ST), mirroring cytokine-responsive fibroblast signatures reported in other single-cell studies (Litvinukova et al., 2020). FB4, less abundant in the right atrium, is characterized by transforming growth factor-β (TGF-β)-responsive genes such as POSTN, whereas FB5 displays high expression of genes driving ECM production and degradation, suggesting specialized matrix-handling roles. These spatial and chamber-biased programs suggest that baseline fibroblast heterogeneity may preconfigure region-specific responses to injury.

Using cellular indexing of transcriptomes and epitopes sequencing (CITE-seq) across 45 human left-ventricle specimens (healthy donor, acute MI, chronic failure), Amrute et al. (2024) identified 13 distinct fibroblast cell states (F1∼13) (Amrute et al., 2024). Notably, F4 (APOE/AGT) and especially F5 (DLK1/GPX3) were relatively enriched in healthy hearts and depleted in disease. Mapping of murine myocardial infarction (MI) and angiotensin II (AngII) datasets to the human reference yielded low similarity scores for F5, suggesting a health-associated state (Amrute et al., 2024).

Beyond simply space occupancy under physiological conditions (Figure 1B), CFs maintain myocardial structure and signaling by coordinating ECM synthesis and turnover (e.g., collagens I/III, fibronectin (FN1), and elastin) (Baudino et al., 2006) and by acting as paracrine signaling hubs that respond to hormonal and inflammatory cues (Lajiness and Conway, 2012; Porter and Turner, 2009). Furthermore, CFs are mechanosensitive, expressing receptors (e.g., integrins) that allow them to sense changes in mechanical load or matrix stiffness and transduce these cues into biochemical responses (Gilles et al., 2020). Another feature that might be overlooked is the electrical and functional coupling between fibroblasts and cardiomyocytes. CFs contribute to the formation of the cardiac skeleton, which electrically insulates the atria from the ventricles (Kleinbongard et al., 2024; Wang et al., 2023). Beyond insulation, CFs engage in direct crosstalk with cardiomyocytes through gap junctions (e.g., Connexin 43 and 45), facilitating not only potential electrical coupling—which can modulate conduction velocity and arrhythmogenesis—but also biochemical coupling allowing for the exchange of small metabolic molecules (Gaudesius et al., 2003). Furthermore, this interaction is mediated physically by the ECM. The stiffness and composition of the matrix synthesized by fibroblasts directly influence cardiomyocyte contractility and mechanotransduction pathways (Frangogiannis, 2019). This tripartite biochemical, electrical, and mechanical crosstalk is critical for maintaining synchronous cardiac function and, when disrupted, plays a central role in the pathogenesis of fibrosis.

### 2.2 Fibroblast subtypes in ischemic cardiac fibrosis

#### 2.2.1 Cellular dynamics post-myocardial infarction: inflammation, fibroblast activation, and scar formation

After MI, necrotic cardiomyocytes release damage-associated molecular patterns (DAMPs), triggering an intense inflammatory cascade (Frangogiannis, 2014) characterized by the infiltration of neutrophils (Puhl and Steffens, 2019) and macrophages (Peet et al., 2020).

In this inflammatory milieu, resident cardiac fibroblasts undergo profound phenotypic changes (Travers et al., 2016). Stimulated by pro-inflammatory cytokines (e.g., tumor necrosis factor alpha, TNF-α and interleukin 1 beta, IL-1β), neurohormones (AngII), and altered mechanical stress in the infarct border zone (BZ), fibroblasts become activated (Porter and Turner, 2009). This activation involves rapid proliferation, with fibroblast numbers expanding significantly within the first week post-MI (Fu et al., 2018). Activated fibroblasts differentiate into myofibroblasts (MYO), a contractile and ECM-producing cell type traditionally marked by the expression of α-smooth muscle actin (α-SMA, Acta2) (Porter and Turner, 2009). MYO are essential for the synthesis of ECM proteins and the formation of a stable infarct scar, which prevents ventricular rupture (Davis and Molkentin, 2014; Talman and Ruskoaho, 2016). However, persistent MYO activation can lead to excessive ECM deposition, contributing to adverse ventricular remodeling, diastolic dysfunction, and arrhythmias (Khan and Sheppard, 2006). A key advance from single-cell profiling is the recognition that “MYO” represents multiple activation states rather than a single uniform population.

#### 2.2.2 Temporal phases of fibroblast response

**FIGURE 2:**
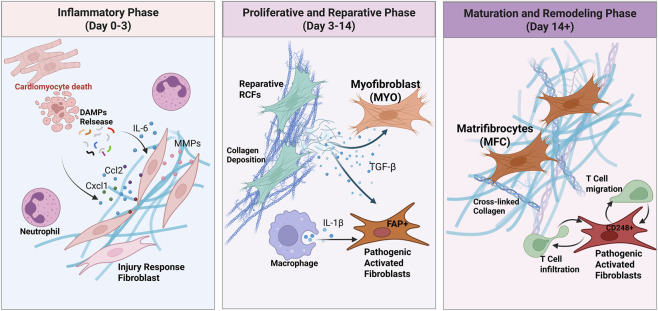
Temporal phases of the cardiac fibroblast response to myocardial infarction. The temporal evolution of fibroblast states following myocardial infarction. The response is categorized into three overlapping phases: Inflammatory Phase (Day 0–3): In response to cardiomyocyte death and the release of DAMPs, fibroblasts adopt a pro-inflammatory “Injury Response” state. These cells secrete cytokines (e.g., IL-6) and chemokines (e.g., Ccl2, Cxcl1) to recruit immune cells such as neutrophils, and express MMPs for initial ECM remodeling. Proliferative and Reparative Phase (Day 3–14): Driven by signals such as TGF-β, fibroblasts differentiate into heterogeneous activated states including Reparative Cardiac Fibroblasts (RCFs) and Myofibroblasts (MYO) that deposit collagen. Macrophage-derived IL-1β may influence the emergence of pathogenic FAP+ activated fibroblasts, which can play a role in maladaptive fibrosis. Maturation and Remodeling Phase (Day 14+): During scar maturation, reparative cells may transition into Matrifibrocytes (MFC), which specialize in maintaining the scar via cross-linked collagen. Conversely, the persistence of CD248^+^ pathogenic fibroblasts can promote T-cell infiltration and migration, contributing to chronic inflammation and ongoing remodeling.

The post-MI response is often conceptualized as three overlapping phases: an initial inflammatory phase, a proliferative/reparative phase, and a maturation/remodeling phase (Figure 2). Fibroblasts traverse distinct transcriptional states across these phases that are shaped by changing microenvironment, giving rise to distinct functional subpopulations (Table 1).

**TABLE 1:** Key fibroblast subtypes.

Subtype name	Key biomarkers	Timeline/peak time	Function
Quiescent fibroblast	PDGFRα, Tcf21	Baseline/Pre-injury	Extracellular matrix (ECM) homeostasis and structural support in the healthy heart
Pro-inflammatory fibroblast	IL6, Ccl2, Cxcl1, MMPs, Mt1/2	Early (Day 1–3)	Not matrix-producing; secretes cytokines/chemokines to recruit immune cells and initiate the inflammatory cascade
Reparative cardiac fibroblast (RCF)	Cthrc1, Postn, Lox, Sox9	Peaks day 7–14	Adaptive repair: Essential for proper collagen synthesis and scar formation to prevent cardiac rupture
Classical myofibroblast	α-SMA (Acta2), Col1a1, Fn1	Proliferative phase (Day 3–14)	Acute repair: ECM deposition and wound contraction to provide initial structural integrity to the infarct
Pathological fibroblast (FAP^+^POSTN^+^)	FAP, POSTN, THY1 (CD90), CD276	Peaks early but persists chronically	Maladaptive fibrosis: Dual role in acute repair and chronic fibrosis, drives ECM remodeling and resembles cancer-associated fibroblasts
Pathological fibroblast (CD248^+^)	CD248, ACKR3	Appears day 7, persists and increases > day 21	Maladaptive fibro-inflammation: Creates a chronic inflammatory niche by retaining T-cells, perpetuating adverse remodeling
Fibroblast-cilp	Cilp	Emerges as a predominant fibrogenic cell type in Ang II-induced hypertension	Drives fibrosis through hypersecretory activity, notably in the absence of significant α-SMA expression
Fibroblast-Thbs4	Thbs4	Emerges as a predominant fibrogenic cell type in Ang II-induced hypertension	Drives fibrosis through hypersecretory activity, notably in the absence of significant α-SMA expression
Matrifibrocyte (MFC)	Col1a1 (high), Acta2(low/negative), Comp, Chad, Cilp2	Maturation/Chronic phase (> day 14)	Scar maintenance: A less active, non-proliferative state responsible for long-term maintenance and slow turnover of the established scar

This table provides a structured overview of the dynamic and heterogeneous fibroblast response, highlighting key populations, molecular identifiers and functions.

##### 2.2.2.1 The inflammatory phase: the pro-inflammatory shift

During the early inflammatory phase, CFs respond to DAMPs and inflammatory cytokines by adopting a pro-inflammatory phenotype (Venugopal et al., 2022). Single-cell studies identify a distinct “Injury Response” fibroblast cluster emerging as early as day 1 post-MI. These cells are characterized by high expression of cytokines (e.g., Interleukin-6, IL6) and chemokines crucial for recruiting immune cells (e.g., C-C motif ligand 2, Ccl2; C-X-C Motif Chemokine Ligand 1, Cxcl1) They also express matrix metalloproteinases (MMPs) that contribute to the initial degradation and remodeling of the ECM. In addition, these fibroblasts express stress-response genes such as Mt1 and Mt2 (metallothioneins), which may protect cells from oxidative stress (Shi et al., 2021). Trajectory analyses suggest this state rapidly evolves towards more activated phenotypes. Pro-inflammatory cytokines like IL-1 is thought to delay the transition to a matrix-producing myofibroblast phenotype, ensuring tissue clearance precedes scar formation (Shinde and Frangogiannis, 2014).

Mechanical stress also assumes a significant regulatory function in this phase. Calcagno et al. leveraged high-resolution Visium spatial transcriptomics on mouse MI models to redefine the border zone based on cardiomyocyte gene expression patterns (Calcagno et al., 2022). Traditionally, the BZ was defined anatomically. Here, gene expression revealed two distinct border zone cardiomyocyte populations, termed BZ1 and BZ2. BZ2 is a thin layer abutting the infarct edge. Cardiomyocytes in this region were distinctly altered, co-expressing Nppa and genes like Xirp2 (a mechanotransduction and cytoskeletal gene). A critical experiment by them confirmed the primacy of mechanical stress in inducing the BZ gene program. They created a “non-ischemic injury” by mechanically needling the myocardium (causing cell loss and mechanical disruption without full ischemia). The result was that BZ2-like gene expression still arose in neighboring cells despite no ischemia, supporting a “loss-of-neighbor hypothesis” (Calcagno et al., 2022). According to this hypothesis, when cardiomyocytes lose their adjacent partners (due to death or physical removal), the sudden mechanical uncoupling triggers a genetic program in the survivors–a program geared towards reinforcing structural integrity and stress signaling. In this scenario, fibroblasts rapidly differentiate into myofibroblasts at sites of mechanical breakage, secreting collagen and proteins like POSTN that form a provisional matrix to hold the tissue together. The spatial co-localization data showed BZ2 cardiomyocytes colocalized with these myofibroblasts (Calcagno et al., 2022), indicating that the fibroblast presence is an inherent part of the border zone identity. Functionally, these fibroblasts likely provide mechanical support (preventing myocardial rupture) and paracrine signals to cardiomyocytes (possibly mitigating overstretch or influencing hypertrophy).

##### 2.2.2.2 The proliferative-reparative phase: a symphony of activated states

As inflammation resolves, the infarct microenvironment shifts toward repair with increased anti-inflammatory signals and growth factors, particularly TGF-β and PDGF (Venugopal et al., 2022; Prabhu and Frangogiannis, 2016). CFs expand markedly (≈2–5-fold within infarct/border zones) (Venugopal et al., 2022), including a transient proliferative state enriched for cell-cycle genes (e.g., marker of proliferation Ki-67, Mki67) (Forte et al., 2022). These cells often co-express early activation markers like POSTN and, in some cells, Ly6a (Park et al., 2019). Concurrently, fibroblasts migrate into the infarct zone (Shinde and Frangogiannis, 2014) and differentiate into multiple MYO-like states that collectively synthesize ECM (Col1a1/Col3a1, FN1; POSTN) and generate contractile forces to consolidate the scar (Lajiness and Conway, 2012). Importantly, single-cell analyses have revealed that high Acta2 expression is not a universal feature of all pro-fibrotic fibroblasts. Many cells that actively deposit matrix may express low or negligible levels of Acta2, indicating that fibroblast activation is a spectrum and that focusing solely on α-SMA as a marker overlooks significant heterogeneity within the cell population (Lendahl et al., 2022).

Single-cell profiling identified an adaptive reparative subtype termed reparative cardiac fibroblasts (RCFs), defined by high collagen triple helix repeat containing 1 (Cthrc1) and co-expression of cartilage oligomeric matrix protein (Comp), and the cross-linking enzyme lysyl oxidase (Lox) (Ruiz-Villalba et al., 2020). RCFs emerge and peak around day 7–14 after MI, localizing almost exclusively within the infarct scar. Their function is essential for survival, as Cthrc1 knockout increases mortality due to ventricular rupture (Ruiz-Villalba et al., 2020). RCF generation involves non-canonical TGF-β signaling and transcriptional regulators including sry-box transcription factor 9 (SOX9), and analogous populations have been reported in swine and human MI tissue (Ruiz-Villalba et al., 2020). By utilizing mouse models with type 2 TGF-β receptor deletion, researchers discovered a significant fatty tissue infiltration within cardiac scars, a condition known as lipomatous metaplasia, suggesting that normally function of TGF-β pathways is important to preserve the identity of reparative fibroblasts (Tuleta et al., 2025).

Conversely, Ninh et al. reported that, in BZ, specific clusters of cells elicit a robust type I interferon (IFN) response, which attenuates the normal fibrotic healing process. While the study characterizes distinct subsets of activated fibroblasts, including proinflammatory (Cxcl5-high) and matricellular (Postn-high) fibroblasts, it does not enumerate a specific “IFN fibroblast.” This absence suggests that the transcriptional influence of the immune microenvironment on cellular identity is a highly localized and specialized phenomenon. Specifically, the data indicate that type I IFN signaling is primarily mediated through a distinct immune niche composed of Ifit1-high monocytes and neutrophils. The IFN signaling pathway exerts suppression upon the normal matricellular program within these niches, wherein the fibroblasts manifest reduced expression of matrix genes (POSTN and Acta2) (Ninh et al., 2024).

CD248^+^ fibroblasts represent a later-appearing activated state associated with chronic inflammation. This population emerges around day 7 after MI, expands to become dominant by approximately day 21, and persists into later stages (up to day 28) in the peri-infarct region (Li et al., 2025; Chen et al., 2025). Mechanistically, CD248 has been linked to a signaling axis involving TGF-β receptors and the atypical chemokine receptor ACKR3, which may promote sustained fibroblast activation and create a niche that retains immune cells, particularly T-cells, thereby driving chronic remodeling (Li et al., 2025).

In human ischemic hearts, CITE-seq identifies a prominent FAP^+^POSTN^+^ fibroblast trajectory associated with both acute MI and chronic ischemic cardiomyopathy (Amrute et al., 2024). These cells express high levels of fibroblast activation protein (FAP), POSTN, THY1 (CD90), and CD276, and their gene programs are enriched for ECM remodeling, cell adhesion, and PI3K-Akt signaling. Their abundance peaks early after an acute MI, contributing to the initial reparative fibrosis. However, unlike a purely adaptive population that would resolve, these cells remain chronically elevated in patients with established ischemic cardiomyopathy, suggesting they may play a dual role in both acute scar formation and the perpetuation of chronic, maladaptive fibrosis. Notably, their transcriptomic signature overlaps with that of cancer-associated fibroblasts, consistent with a highly activated, niche-shaping state (Amrute et al., 2024).

The discovery and characterization of these diverse activated states provide profound clarity on the dual nature of cardiac fibrosis. The process is not uniformly “good” or “bad”. Rather, these opposing characteristics can be mapped onto distinct fibroblast subtypes. The CTHRC1^+^ RCFs represent the adaptive face of fibrosis, performing the essential task of building a life-saving scar. In contrast, the persistent FAP^+^POSTN^+^ and CD248^+^ subtypes represent the maladaptive face, driving chronic inflammation and progressive fibrosis. This distinction is the cornerstone of developing precision anti-fibrotic therapies.

##### 2.2.2.3 Maturation-remodeling phase: matrifibrocytes and chronic pathological states

Following the initial proliferative phase of cardiac repair, a critical transition to scar maturation occurs. This involves ECM cross-linking and deactivation or apoptosis of reparative myofibroblasts (Prabhu and Frangogiannis, 2016). This transition is promoted by withdrawal and active counter-regulation of fibrogenic cues (notably TGF-β and AngII), including inhibitory Smads such as Smad7 (Humeres et al., 2022). Similarly, the activation of the angiotensin II type 2 (AT2) receptor can counteract the pro-fibrotic effects mediated by the type 1 (AT1) receptor (Castoldi et al., 2021). Additional “STOP” signals and anti-fibrotic mediators (e.g., prostaglandins) further promote MYO quiescence and termination of the proliferative program (Frangogiannis, 2014; Stratton and Shiwen, 2010). Nevertheless, during this phase, a moderate level of TGF-β signaling might be requisite. Tuleta et al. demonstrated that the loss of TGF-β signaling caused CFs in the infarct zone transdifferentiate into adipocytes, resulting in extensive infiltration of the infarct with adipose tissue 28 days after MI, altering the structural composition of the scar and potentially impacting myocardial stability (Tuleta et al., 2025).

With declining pro-fibrotic signaling, activated fibroblasts may undergo apoptosis or transition into matrifibrocytes (MFC), thought to derive from MYO that downregulate Acta2 and exit the cell cycle (Shi et al., 2021). MFCs retain high ECM gene expression (e.g., Col1a1) but show reduced contractile signatures and upregulate cartilage- and stable-matrix genes (Comp, Chondroadherin (Chad), and cartilage intermediate layer protein 2 (Cilp2)), consistent with long-term scar maintenance (Shi et al., 2021; Torimoto et al., 2024). The persistent FAP^+^POSTN^+^ population identified in human hearts also shares features with MFC, suggesting they may represent a human correlate of this late-stage, scar-resident cell (Amrute et al., 2024).

A persistent activation state, particularly of CD248^+^ fibroblasts, may contribute to ongoing remodeling. Failure to appropriately deactivate MYO and resolve inflammation can result in a chronic fibrotic response, characterized by continued ECM deposition and the expansion of fibrosis into the border and remote zones, ultimately leading to progressive cardiac dysfunction. This concept of “perpetual wound healing” provides a framework for understanding the transition from acute repair to chronic heart failure.

Overall, sequential microenvironmental cues (DAMPs, cytokines, growth factors, evolving matrix signals) drive transitions among inflammatory, proliferative, reparative, and maturation-associated fibroblast states, underscoring the limitations of single markers such as α-SMA and motivating transcriptomic and state-based classification (Table 1).

### 2.3 Cardiac fibroblast heterogeneity in pressure overload-induced fibrosis

In the context of chronic pressure overload, resulting from conditions like systemic hypertension or aortic stenosis, the predominant fibrosis pattern is reactive interstitial and perivascular fibrosis-diffuse ECM accumulation in the relative absence of massive cardiomyocyte death (Travers et al., 2016; Creemers and Pinto, 2011; Chimenti et al., 2025). This contrasts with post-MI replacement fibrosis, where an acute scar is required for mechanical integrity but later contributes to dysfunction and is often accompanied by reactive fibrosis in remote myocardium (Talman and Ruskoaho, 2016; Karur et al., 2024). Accordingly, therapeutic strategies must distinguish adaptive repair from chronic, potentially reversible reactive fibrosis.

#### 2.3.1 The hemodynamic and cellular triggers

The primary driver of pressure overload-induced reactive fibrosis is sustained mechanical stress on the myocardium. Increased wall tension is sensed by cardiac fibroblasts through mechanosensitive receptors, ion channels, and integrins, leading to the activation of signaling pathways that promote ECM production and fibroblast activation (Diez, 2007). Yes-associated protein (YAP) and transcriptional coactivator with PDZ-binding motif (TAZ) are mechanosensitive transcriptional coactivators within the Hippo signaling pathway (Islam and Hong, 2024). Mechanotransduction processes influence Yap/Taz localization and activity, linking the pathway to tissue mechanics and organ fibrogenesis (Chu and Quan, 2024; He et al., 2022). Garoffolo et al. showed that YAP regulates TGF-β1 to impact the fibrotic programming of cardiac stromal cells (Garoffolo et al., 2022). Another investigation reveals that in response to a rigid matrix, CFs augment YAP expression and nuclear localization, resulting in cell activation (Niu et al., 2020). *In vivo* studies also demonstrated that increased wall stress directly correlates with increased collagen synthesis and reduced activity of collagen-degrading enzymes (Diez, 2007). Using multiple transgenic murine models, He et al. demonstrated that early injury-induced myofibroblast YAP/TAZ activation is a key event driving fibrosis in multiple organs (He et al., 2022). Notably, it has been shown that fibroblast-specific deletion of YAP/TAZ can reduce MI-induced cardiac fibrosis (Mia et al., 2022). Additionally, integrin α11 (Itga11) is one of the collagen-binding integrins expressed on cardiac fibroblasts and has been implicated in mechanotransduction and fibrotic responses in cardiac tissue (Civitarese et al., 2016). Itga11’s engagement with collagen appears to regulate cellular collagen assembly, mechanosensing, and downstream fibrotic pathways (e.g., NF-κB signalling) (Romaine et al., 2018), and recent work also shows that transcriptional regulators (e.g., TFAP4) directly upregulate Itga11 to enhance cardiac fibrosis by augmenting fibroblast mechanotransduction and ECM gene expression (Liu et al., 2025).

This mechanical stimulus is amplified by the activation of neurohormonal systems, most prominently the renin-angiotensin-aldosterone system (RAAS) (Hu et al., 2024). AngII, a key effector of the RAAS, is a potent pro-fibrotic agent that acts directly on CFs, binding to its AT1 receptor to stimulate proliferation, differentiation, and the synthesis of ECM components (Garvin et al., 2021).

These mechanical and neurohormonal insults converge on a state of chronic, low-grade inflammation, which perpetuates the fibrotic response (Frangogiannis, 2021). Pro-fibrotic cytokines and growth factors, particularly TGF-β, are master regulators of this process (Garvin et al., 2021). Released by various cells including macrophages and the fibroblasts themselves, TGF-β promotes the differentiation of quiescent fibroblasts into activated, ECM-secreting MYO (Garvin et al., 2021). Specifically, Septin4 has emerged as a critical regulator of cardiac fibrosis in this context (Yucel et al., 2025). Predominantly expressed in CFs and endothelial cells, Septin4 is required for the production of structural proteins like collagen and cross-linking enzymes. Its absence results in reduced ECM density and increased cardiac compliance both at baseline and following pressure overload injury. Mechanistically, Septin4 is necessary for effective TGF-β signaling, as Septin4-deficient fibroblasts exhibit reduced TGF-β receptor 1 (TGFBR1) expression, blunted Smad2 phosphorylation, and a failure to upregulate profibrotic genes upon stimulation.

Fibroblast activation is not driven only by cytokines (e.g., TGF-β) but are also enabled and stabilized by shifts in core metabolism and by metabolite signaling that rewires gene expression through receptor pathways and metabolite-sensitive enzymes or epigenetics (Gibb et al., 2020). Recently, lactate has been linked to histone lactylation in fibroblasts as an additional transcriptional control layer that can amplify pro-fibrotic programs (including TGF-β1 expression in atrial fibroblasts), illustrating a direct metabolic-epigenetic route to fibroblast activation (Fang et al., 2025). In parallel, TCA-cycle metabolites act as regulators rather than mere intermediates. For example, α-ketoglutarate (α-KG) is a required cofactor for prolyl hydroxylases that control HIF-1α stability and for α-KG–dependent chromatin enzymes, linking oxygen/metabolic state to gene programs relevant to activation. In cardiac remodeling models, α-KG supplementation has been reported to attenuate pressure-overload remodeling and fibrosis, consistent with metabolite-level modulation of fibrotic outcomes (An et al., 2021). Finally, succinate, often elevated in hypoxia/inflammation, can drive fibroblast activation in aged mice and humans by enhancing fibroblast activation and collagen production through PKM2 succinylation–dependent remodeling of glycolytic control (Wang et al., 2025).

This intricate interplay between mechanical forces, neurohormonal pathways, metabolite signaling and inflammatory mediators creates a self-sustaining cycle that drives the relentless progression of reactive fibrosis (Maruyama and Imanaka-Yoshida, 2022).

#### 2.3.2 The source of fibrosis

The origin of fibroblasts accumulating during fibrosis was long debated. Earlier models proposed substantial recruitment from circulating fibrocytes or adult EndoMT, but conclusions were confounded by non-specific markers shared with immune, endothelial (via EndoMT), and perivascular cells (Talman and Ruskoaho, 2016). In certain pathological contexts, current evidence supports that EndoMT contributes to the generation of fibroblast-like cells that participate in cardiac fibrosis. Recent study highlight that aging and related endothelial dysfunction are associated with enhanced EndoMT activation, contributing to cardiovascular fibrosis and the decline in vascular homeostasis that accompanies aging (Singh et al., 2024). In parallel, pericytes in the healing infarct can adopt fibrogenic programs. In a mouse MI model, pericytes diversified into matrix-synthetic or matrix-remodeling states, and a small fraction became pericyte-derived fibroblasts that expressed higher levels of structural collagens and matricellular genes compared with fibroblasts from other sources—supporting a disproportionate ECM-remodeling contribution despite low abundance (Alex et al., 2023). Vascular smooth muscle cells (VSMCs) also have ECM-producing capacity under pathological stress (often described as a “synthetic” shift) and are discussed as key ECM-remodeling cells in cardiovascular fibrotic remodeling, particularly in perivascular compartments (Bachmann et al., 2022). However, in pressure-overload remodeling, inducible fate-tracing with single-cell mapping suggests that the major expansion toward ECM-expressing programs occurs in fibroblast populations, emphasizing that non-fibroblast contributions may be more context- and niche-restricted than fibroblast-driven interstitial fibrosis (Peisker et al., 2022).

Rigorous genetic lineage tracing using fibroblast-lineage markers (PDGFRα, Tcf21) and reporters (e.g., Col1a1-GFP) indicates that pressure-overload fibrosis is driven predominantly by proliferation/activation of pre-existing resident cardiac fibroblasts (Reichardt et al., 2021; Aguado-Alvaro et al., 2024; Bursac, 2014). In transverse aortic constriction (TAC) models, both epicardium-derived and endocardium-derived fibroblast pools proliferate while maintaining their spatial distributions, with little contribution from hematopoietic cells, circulating progenitors, or adult EndoMT (Moore-Morris et al., 2014). This shifts therapeutic attention toward modulating resident fibroblast activation and expansion rather than blocking external recruitment.

Another Frontier in cardiac fibroblast research is understanding the epigenetic and transcriptional regulatory mechanisms that underlie fibroblast state transitions. Fibroblast activation involves widespread changes in chromatin accessibility and gene expression, orchestrated by transcription factors (TFs) and chromatin-modifying complexes. A large-scale CRISPR screen targeting 750 chromatin regulators in primary cardiac fibroblasts identified dozens of epigenetic modifiers that govern fibrotic transformation (Aguado-Alvaro et al., 2025). Notably, several histone acetylation complexes (the Tip60/KAT5 complex, SRCAP complex, and NSL complex) emerged as pro-fibrotic regulators–when these were knocked out, fibroblasts were less able to downregulate PDGFRα and become TGF-β-induced myofibroblasts. In contrast, components of the Polycomb repressive complex (e.g., EZH2) and certain chromatin remodelers (e.g., BRG1/BRD9) acted as anti-fibrotic brakes, whose loss made fibroblasts hyper-responsive to TGF-β. Mechanistically, it appears that deposition of the histone variant H2A.Z by SRCAP and its acetylation by Tip60 create a more permissive chromatin state at fibrosis gene loci, allowing pro-fibrotic TFs (like SMAD3, SRF, etc.) to bind and activate transcription. Disrupting this epigenetic switch (for instance with a Tip60 inhibitor) has been proposed as a novel anti-fibrotic strategy (Aguado-Alvaro et al., 2025). In parallel, transcription factor network analysis in single-cell studies has highlighted TFs associated with specific fibroblast subtypes. For example, one analysis found that within the myofibroblast population there were two subclusters–a profibrotic subcluster with higher VDR, GLI2, and SMAD3 activity, and an anti-fibrotic subcluster with higher ESR1 (estrogen receptor) and SMAD1/5 activity. This hints that differential TF activation can bias fibroblasts toward more scar-promoting versus scar-resolving phenotypes (Patrick et al., 2024). Finally, developmental studies suggest fibroblasts retain some of their original transcriptional identity–for instance, subsets of adult fibroblasts still express Tbx18 or Wt1 long after development, and these genes may subtly influence their behavior (Deng et al., 2023). Cutting-edge functional genomics and epigenomic profiling are revealing that fibroblast heterogeneity is underpinned by distinct transcriptional programs and chromatin landscapes. Understanding these programs not only explains how a fibroblast “decides” to become a myofibroblast vs. a pro-reparative fibroblast, but also offers opportunities to intervene by reprogramming harmful fibroblast states at the gene regulation level.

#### 2.3.3 Novel disease-associated subtypes identified by scRNA-seq

High-throughput scRNA-seq enables unbiased profiling of fibroblast states beyond marker-defined categories and has expanded catalogs of disease-associated fibroblast programs and regulatory pathways (Fuster-Martinez and Calatayud, 2024) (Table 1). One of the most significant discoveries is a pathogenic fibroblast population characterized by high expression of FAP and POSTN in both human and animal models of fibrosis (Amrute et al., 2024). This FAP^+^POSTN^+^ fibroblast lineage is considered particularly pathogenic, as it is associated with chronic remodeling and a pro-fibrotic inflammatory niche. The fact that this subtype appears across different etiologies of fibrosis suggests it may represent a common therapeutic target (Amrute et al., 2024). Additionally, inflammatory fibroblast subtypes have been described that express cytokines and chemokines such as chemoattractant protein-1 (MCP-1) and IL-6. These fibroblasts are thought to play a role in recruiting and interacting with immune cells, thereby contributing to the chronic inflammatory state that fuels fibrosis (Trial et al., 2016).

#### 2.3.4 Myofibroblast and matrifibrocytes in pressure overload models

While MFC are traditionally defined as a resolution-stage subpopulation derived from myofibroblasts in replacement fibrosis (such as after a myocardial infarction), evidence from pressure overload models indicates that they can arise through a distinct lineage (McLellan et al., 2020). In the context of AngII-induced hypertensive cardiac injury, single-cell transcriptomics and lineage tracing reveal that quiescent fibroblasts differentiate directly into ECM-producing cells (Fibroblast-Cilp and Fibroblast-Thbs4). These cells exhibit transcriptomic and functional signatures congruent with a MFC identity, yet notably, this phenotypic transition occurs independently of a transient α-SMA^+^ MYO intermediate (McLellan et al., 2020). Interestingly, a recent scRNA-seq study in AngII hearts also suggested that the classical α-SMA^+^ MYO may not be the dominant cell type driving fibrosis in pressure overload (Patrick et al., 2024). However, immunofluorescence analysis of mid-ventricular sections revealed that fibrotic remodeling at day 7 was characterized by the focal emergence of large tdTomato^+^α-SMA^+^ cardiac fibroblasts. These activated myofibroblasts were localized within interstitial and periarteriolar niches, coinciding with robust collagen deposition. While tdTomato-labeled fibroblasts persisted in these regions at day 14, they exhibited a marked downregulation of α-SMA expression (low-to-negative), a phenotypic profile indicative of matrifibrocytes (Patrick et al., 2024). These temporal dynamics delineate a transient wave of myofibrogenesis following AngII-induced stress that largely resolves within 2 weeks. Based on these data, researchers hypothesize that the MFC present in the late stage of cardiomyopathy are lineage-derived from the early-stage myofibroblast pool (Patrick et al., 2024). These findings underscore the importance of context-specific fibroblast trajectories and indicate that anti-fibrotic therapies may need to be tailored to the specific type and stage of cardiac injury.

## 3 The critical dialogue: immune-fibroblast crosstalk

The macrophage-IL-1β-fibroblast axis provides a mechanistic link between inflammation and fibrosis (Alexan et al., 2024). The process begins when cardiac stress triggers the activation of myeloid cells, particularly macrophages. Through chromatin-level changes involving the bromodomain protein BRD4, these activated macrophages upregulate and secrete IL-1β (Alexan et al., 2024). CFs expressing the IL-1β receptor (IL1R1) respond through NF-κB (p65/RELA) to induce MEOX1, a regulator of pathogenic activation that promotes differentiation toward the pro-fibrotic FAP^+^POSTN^+^ program (Amrute et al., 2024; Alexan et al., 2024).

Furthermore, a recent study identified a metabolic-epigenetic axis where cardiac macrophages undergo significant metabolic reprogramming, marked by increased *de novo* fatty acid synthesis and elevated levels of key enzymes, including ATP citrate lyase (ACLY) and fatty acid synthase (FASN) following MI. ACLY cleaves citrate to produce acetyl-CoA, which serves as the essential substrate for histone acetyltransferases within the nucleus. This activity promotes H3K27 acetylation—a mark of active gene promoters—in the promoter regions of key pro-fibrotic genes, including Krt17, Il33, and Nlrp3, thereby priming them for active transcription. This epigenetic reprogramming in macrophages drives a paracrine signaling environment that promotes the expansion of the pathogenic fibroblast population, exacerbating fibrosis (Sadaf et al., 2025). In addition, recent literature supports that interleukin-11 (IL-11) is a key mediator of fibroblast activation. In primary human fibroblasts exposed to TGF-β1, IL-11 upregulation is consistently one of the strongest transcriptional responses, and both IL-11 and its receptor IL11RA are expressed in fibroblasts, enabling an autocrine pro-fibrotic signaling loop. This IL-11 signaling drives excessive synthesis of ECM components such as collagen, and neutralizing IL-11 or deleting IL11RA in mice confers significant protection against fibrosis in models of pressure-overload and ischemic injury, indicating its functional importance in cardiac fibrogenesis (Schafer et al., 2017).

By framing fibrosis as a specified consequence of discrete inflammatory signals, these findings suggest that blocking IL-1β/IL-11 signaling (or their upstream production) could prevent emergence of the most pathogenic fibroblast lineage and uncouple inflammation from fibrotic remodeling.

While macrophage-fibroblasts interactions are prominent initially, chronic progression appears to involve a different arm of the immune system. Lymphocytes are critical drivers of cardiac fibrosis, infiltrating the myocardium in a biphasic manner following injury to regulate fibroblast phenotype and scar formation. This regulation is bidirectional- while lymphocytes secrete cytokines that modulate fibroblast activity, cardiac fibroblasts can also act as antigen-presenting cells expressing MHC-II to directly activate T cells and perpetuate the immune response. The lymphocyte-fibroblast crosstalk has recently been well-reviewed by Learmonth et al. (2023). Briefly, CD4^+^ T helper cells infiltrate the myocardium in a biphasic manner and exert divergent, subset-specific control over fibroblast function and cardiac remodeling. Th1 cells primarily act as anti-fibrotic regulators by secreting Interferon-γ (IFN-γ), which inhibits TGF-β signaling and blocks myofibroblast differentiation. However, they also secrete IL-2, which can conversely stimulate fibroblast proliferation and activation via JAK-STAT pathways. In the chronic phase, Th2 cells predominate and promote fibrosis by secreting IL-4 and IL-13, cytokines that suppress Th1 activity and facilitate collagen deposition, often indirectly through the polarization of M2 macrophages. Regulatory T cells (Tregs) generally limit fibrosis and maintain homeostasis by dampening inflammation and directly reducing fibroblast α-SMA expression, though they exhibit plasticity and can transition to a dysfunctional, pro-fibrotic phenotype in chronic heart failure (Learmonth et al., 2023).

Late-activated CD248^+^ fibroblasts were found to be spatially co-localized with CD3^+^ T cells in the peri-infarct region of both mouse and human hearts. These fibroblasts were shown to promote the retention and activation of T cells, contributing to a chronic inflammatory state. Indeed, genetic deletion of Cd248 in mice led to a significant reduction in cardiac T-cell infiltration following ischemic injury (Li et al., 2025). This suggests that CD248^+^ fibroblasts play a key role in shaping the immune landscape of the chronically injured heart. Moreover, these T cells, in turn, can secrete cytokines that further stimulate fibroblast activation and ECM production, creating a pathological feed-forward loop that sustains inflammation and fibrosis.

Mechanistically, CD248 does not appear to act as a direct ligand for a T-cell receptor. Instead, it functions intracellularly to stabilize a key signaling component (protecting it from lysosomal degradation), thereby augmenting downstream signaling and promoting upregulation and stabilization of ACKR3 on the fibroblast surface. Increased ACKR3 expression is proposed to enhance adhesion and retention of T-cells within the fibrotic niche (Li et al., 2025).

Recent literature indicates that interferon pathways are actively engaged in injured hearts and can reprogram cardiac fibroblasts toward interferon-responsive, immune-modulatory states with implications for fibrotic remodeling. Using mouse and human MI datasets, a key study showed that stressed BZ cardiomyocytes initiate type I interferon colonies (IFNICs), composed of cells expressing interferon stimulated genes (ISGs) such as Ifit1, Ifit3, and Cxcl10, and reported that interferons blunt protective matricellular programs and contractile function of fibroblasts, increasing vulnerability to pathological remodeling and rupture (Ninh et al., 2024). Complementing this, a integrative single-cell map of cardiac fibroblasts across diverse fibrotic pathologies consistently recovered a rare “fibroblast–interferon stimulated” (F-IFNS) population with a strong type I IFN-stimulated gene signature that is present across the MI time course, supporting the idea that interferon signaling defines a distinct inflammatory fibroblast state rather than being confined to leukocytes (Patrick et al., 2024). Mechanistically, a study further demonstrated that IFN-β–IFNAR–pSTAT1 signaling in CFs can induce chemokines (e.g., CCL2/7/12) that recruit IFN-β–secreting macrophages, forming a reinforcing inflammatory circuit that reshapes fibroblast behavior in the post-MI microenvironment (Wang et al., 2024). These fibroblast-centered findings align with earlier causal evidence that excessive type I IFN signaling (IRF3/IFNAR axis) worsens post-MI outcomes *in vivo* (King et al., 2017). In TAC model, Fabian et al. also identified an IFN fibroblast subtype (IntFib; Ifit1^+^, Ifit3^+^), which showed specific upregulation of ISGs (Peisker et al., 2022). Together, the current view is context-dependent but convergent: interferons can impose interferon-stimulated, chemokine-secreting fibroblast states and, in the MI border zone, may directly suppress reparative fibroblast programs—both routes that can plausibly tilt remodeling toward maladaptive fibrosis or scar dysfunction.

This reciprocal interaction between immune cells and fibroblast subtypes highlights that fibrosis is not merely an overproduction of ECM but a complex process orchestrated by dynamic cellular communication. Understanding and targeting these specific immune-fibroblast signaling axes offers a promising strategy for developing more effective and precise anti-fibrotic therapies.

## 4 Therapeutic implications: precision targeting of pathogenic fibroblast subtypes

### 4.1 The rationale for targeting specific fibroblast subpopulations

Cardiac fibrosis contributes to dysfunction, arrhythmias, and heart-failure progression, yet no approved therapies directly target the fibrotic process or effectively reverse established fibrosis (Diez et al., 2020). This gap motivates approaches that intervene more specifically in the cellular programs that sustain fibrotic remodeling (Diez et al., 2020).

The paradigm shift from viewing fibroblasts as a homogeneous population to recognizing their heterogeneity has profound implications for therapeutic development. Traditional anti-fibrotic strategies have largely focused on broadly inhibiting pro-fibrotic signaling pathways, such as TGF-β, with limited success and often significant off-target effects. The new understanding suggests that targeting specific pathogenic fibroblast subtypes that drive maladaptive fibrosis, while sparing fibroblasts involved in normal homeostasis and essential repair, could provide a more effective and safer approach (Emig et al., 2019). This precision medicine strategy aims to eliminate or reprogram the “bad” fibroblasts without disrupting the beneficial functions of the “good” ones.

Several specific fibroblast subpopulations have been proposed as key therapeutic targets based on their association with disease and their distinct molecular markers. These include the chronic FAP^+^POSTN^+^ fibroblasts, as well as other subtype-specific markers identified in different disease contexts, such as the AEBP1^+^ fibroblasts in hypertrophic cardiomyopathy, Lox^+^ fibroblasts in diabetic cardiomyopathy, and Thbs4^+^ fibroblasts in pressure overload models (Kattih et al., 2023; Li et al., 2023; Dewar et al., 2024). The identification of cell surface markers and molecular signaling unique to these subtypes provides a means for selectively targeting them with advanced therapies, such as antibody-drug conjugates or engineered immune cells.

### 4.2 Challenges in developing subtype-specific anti-fibrotic therapies

Translating fibroblast heterogeneity into safe therapies remains challenging. A key hurdle is identifying targets that are sufficiently enriched in pathogenic cardiac fibroblast states while minimizing expression in quiescent CFs, other cardiac lineages, and fibroblasts in essential organs (Humeres and Frangogiannis, 2019; Micheletti and Alexanian, 2022). Other challenges include the inherent plasticity of fibroblasts, which might allow them to switch phenotypes or develop resistance to targeted therapies (Kleinbongard et al., 2024). The optimal timing for intervention is also unclear. Targeting too early might disrupt necessary repair, while targeting too late might be ineffective against established, cross-linked fibrosis (Micheletti and Alexanian, 2022). Furthermore, translating findings from preclinical animal models, where much of the heterogeneity research is conducted, to the complexity of human heart disease requires careful validation (Shi et al., 2021). Finally, advanced therapeutic modalities like cell therapies or RNA-based drugs face practical barriers in delivery, stability, immunogenicity, and cost (Micheletti and Alexanian, 2022).

### 4.3 Targeting pathogenetic fibroblast subtypes

The detailed elucidation of fibroblast heterogeneity has opened a new Frontier for the development of anti-fibrotic therapies. Among proposed targets, the FAP^+^ activated fibroblast state is attractive due to the unique expression pattern of fibroblast activation protein (Cui et al., 2023). FAP is a type II transmembrane serine protease that is highly expressed on the surface of activated fibroblasts in areas of active fibrosis and tumor stroma (Roy et al., 2020; Fitzgerald and Weiner, 2020). This remarkable specificity makes FAP an ideal target for both diagnosis and therapy.

**FIGURE 3:**
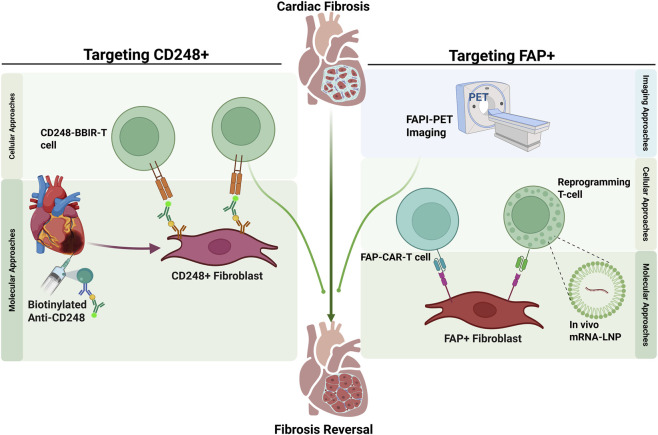
Targeted Immunotherapeutic Strategies for Reversing Cardiac Fibrosis. The schematic outlines two advanced therapeutic approaches for reversing cardiac fibrosis through the selective depletion of pathogenic fibroblast subsets. Left Panel (Targeting CD248^+^ Fibroblasts): Depicts a two-step safety-enhanced strategy combining molecular and cellular approaches. First, a biotinylated anti-CD248 antibody is locally injected into the fibrotic heart tissue to specifically tag CD248^+^ fibroblasts. Subsequently, CD248-BBIR-T cells (Biotin-Binding Immune Receptor-engineered T cells) are administered. These cells are designed to activate strictly upon recognition of the biotin tag, ensuring precise, spatially and temporally controlled depletion of the target fibroblasts to minimize off-target effects. Right Panel (Targeting FAP^+^ Fibroblasts): Illustrates a theranostic strategy. Imaging Approach: FAP inhibitors for non-invasive Positron Emission Tomography (FAPI-PET) imaging utilizes radiolabeled FAP inhibitors to non-invasively visualize and quantify active fibrosis prior to treatment. Cellular and Molecular Approaches: Therapy involves the elimination of FAP^+^ fibroblasts via FAP-CAR-T cells. Two methods for generating these therapeutic cells: direct infusion of engineered CAR-T cells or *in vivo* reprogramming of endogenous T-cells using mRNA-LNP (lipid nanoparticles) to induce temporary CAR expression.

The development of small molecule FAP inhibitors (FAPI) conjugated to positron-emitting radionuclides (e.g., [68Ga]Ga-FAPI-04) has given rise to FAPI-PET imaging (Chandekar et al., 2023). This non-invasive imaging modality allows for the direct visualization and quantification of FAP-expressing activated fibroblasts in the heart (Figure 3). FAPI-PET may detect active fibrogenesis earlier than LGE-MRI (which largely reflects established scar) (Bing and Dweck, 2019), and early studies link cardiac FAPI uptake with risk factors and adverse remodeling, supporting patient stratification and therapy monitoring (Cui et al., 2023).

FAP can also be therapeutically targeted. In mouse hypertensive cardiac injury, a single infusion of FAP- chimeric antigen receptor (CAR)-T cells infiltrated the heart, eliminated FAP^+^ fibroblasts, reduced established fibrosis, and improved systolic and diastolic function (Aghajanian et al., 2019). Furthermore, Rurik et al. further showed that CD5-targeted lipid nanoparticles (LNP) delivering modified mRNA encoding the same anti-FAP CAR can transiently reprogram host T cells *in vivo*. These mRNA-generated CAR-T cells homed to fibrotic myocardium and reversed fibrosis and dysfunction in mice (Rurik et al., 2022). Together, FAP-targeted imaging and therapy suggest a “theranostic” paradigm in which FAPI-PET identifies patients with active FAP^+^ fibrogenesis and guides subtype-directed intervention (Figure 3).

Targeting CD248-enriched in late, chronically inflammatory fibroblasts-offers a strategy to preserve early reparative fibrosis while limiting chronic progression (Li et al., 2025). CD248-CAR-T cells eliminated CD248^+^ fibroblasts, reduced T-cell infiltration, attenuated fibrosis, and improved function in mouse MI models (Li et al., 2025). A switchable Biotin-Binding Immune Receptor (BBIR)-T platform adds spatial and temporal control (Figure 3) (Chen et al., 2025). Biotinylated anti-CD248 fragments “paint” target cells via intramyocardial delivery, and intravenously infused BBIR-T cells are activated only upon encountering biotin-labeled targets. In preclinical studies, BBIR-T therapy cleared CD248^+^ fibroblasts and limited fibrosis expansion while minimizing systemic inflammation and off-target injury (Chen et al., 2025). These cell-based therapies, particularly those targeting late-stage markers like CD248 with built-in safety switches, represent a future direction for treating chronic cardiac fibrosis.

Despite their transformative potential, these T-cell approaches have notable caveats. Unlike the permissive environments of hematologic malignancies, the heart’s microvascular endothelium and evolving fibrotic architecture can plausibly impede cell migration and spatially restrict T cells access (especially into dense scar tissue) (Prabhu and Frangogiannis, 2016). The FAP-CAR strategies risk collateral depletion of reparative myofibroblasts, emphasizing the need for state-specific antigen selection. In addition, FAP is low but not completely absent in some normal human cells. For example, FAP has been identified on multipotent bone marrow stromal cells (mesenchymal stem cells) where it serves as a surface marker in adults, suggesting a role beyond pathology (Chung et al., 2014). Consequently, the potential for “on-target, off-fibroblast” toxicity remains a salient concern that warrants rigorous investigation. The mRNA-LNP reprogramming avoids *ex vivo* cell manufacture but may involve non-specific LNP uptake, transient CAR expression requiring repeat dosing, and limited large-animal validation. The CD248-BBIR approaches improve controllability but require a technically demanding two-step protocol (intramyocardial biotin-antibody plus timed systemic T-cell infusions) and may face durability and immunogenicity constraints with repeated adaptor and antibody exposure.

### 4.4 Blocking immune-fibroblast cell cross-talk in cardiac fibrosis

**FIGURE 4:**
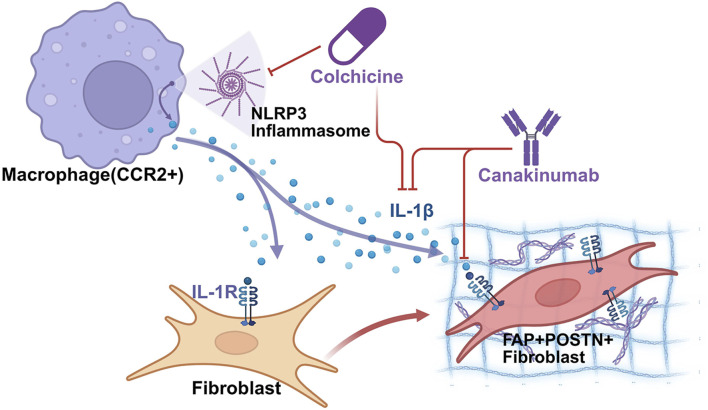
The Macrophage-Fibroblast IL-1β Signaling Axis as a Therapeutic Target in Cardiac Fibrosis. This schematic illustrates the mechanism by which immune cells drive pathological tissue remodeling and identifies specific pharmacological intervention points. Macrophage Activation: The process initiates with activated macrophages (CCR2^+^), which assemble the intracellular NLRP3 inflammasome. This complex facilitates the processing and secretion of the pro-inflammatory cytokine Interleukin-1β (IL-1β, represented by blue spheres). Fibroblast Differentiation: Secreted IL-1β binds to the IL-1 receptor (IL-1R) on the surface of quiescent fibroblasts. This signaling event acts as a primary switch (indicated by the curved arrow), driving the transition of fibroblasts into an activated, pro-fibrotic phenotype. Pathological Outcome: The activated fibroblasts are characterized by the upregulation of Fibroblast Activation Protein (FAP) and Periostin (POSTN). These cells are responsible for the excessive production and deposition of extracellular.

An alternative to directly killing pathogenic fibroblasts is to target the signaling pathways that drive their differentiation and activation (Figure 4). As established, IL-1β from macrophages is a key driver of the FAP^+^POSTN^+^ pathogenic fibroblast lineage (Amrute et al., 2024). The clinical relevance of this pathway was suggested by the CANTOS trial (Canakinumab Anti-inflammatory Thrombosis Outcomes Study) (Ridker et al., 2017). In this trial, IL-1β blockade with canakinumab reduced major cardiovascular events in post-MI patients with elevated high-sensitivity C-reactive protein (hsCRP) (HR 0.85; 95% CI 0.74–0.98; P = 0.021). It is worth noting that CANTOS did not directly assess myocardial fibrosis, and that reported benefits were cardiovascular event–related rather than fibrosis endpoints. Mechanistically, spatial and chromatin analyses implicate IL-1β signaling from CCR2^+^ macrophages to FAP^+^POSTN^+^ fibroblasts as a central pathway promoting fibrotic remodeling (Figure 4). In mouse models (TAC, AngII infusion, and post-infarction remodeling), anti-IL-1β antibody treatment reduced FAP^+^POSTN^+^ fibroblasts, decreased collagen content, and improved left-ventricular function (Amrute et al., 2024). Furthermore, Alexanian et al. also shown that macrophage-restricted IL-1β deletion (Cx3cr1 lineage) produced similar protection (Alexan et al., 2024).

Because NLRP3 inflammasome activity contributes to IL-1β production, its inhibition is another potential strategy (Floris et al., 2023). CANTOS subanalysis suggested residual interleukin-18 (IL-18) and interleukin-6 (IL-6) inflammation in some patients despite IL-1β blockade, motivating broader inflammasome or combinatorial approaches (Potere et al., 2024). Consistently, colchicine-which can suppress IL-1β/IL-18/IL-6 via NLRP3 inhibition-has shown benefit across trials (Ridker et al., 2020; Nidorf et al., 2020; Tardif et al., 2019). A meta-analysis encompassing five randomized controlled trials with a total of 11,816 patients diagnosed with atherosclerotic cardiovascular disease demonstrated that long-term administration of low-dose colchicine is associated with a 22% reduction in major adverse cardiovascular events (Fiolet et al., 2021).

Given the central role of TGF-β in fibrosis, significant research efforts are focused on targeting its signaling pathway using antibodies, small molecule inhibitors, or by interfering with the mechanisms that activate latent TGF-β (Parichatikanond et al., 2020). In PIROUETTE trail, pirfenidone reduced an MRI-based fibrosis marker in HFpEF but did not improve exercise capacity or quality of life, underscoring the challenge of translating biomarker changes into clinical benefit (Lewis et al., 2021).

## 5 Discussion

The research landscape of cardiac fibrosis has undergone a profound transformation. The once-monolithic CFs is now recognized as a remarkably heterogeneous population with distinct subtypes that execute specialized (and sometimes opposing) functions during disease evolution (Luo et al., 2024). Despite this remarkable progress, critical questions remain.

### 5.1 A conceptual framework for fibroblast states

**FIGURE 5:**
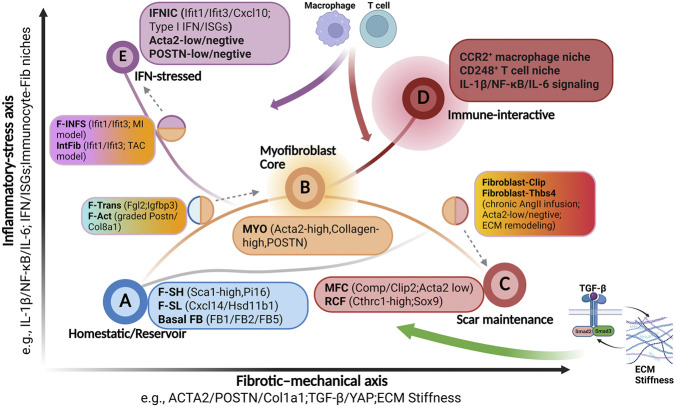
Conceptual framework Visualization of Cardiac Fibroblast States. This conceptual framework visualizes cardiac fibroblast heterogeneity as a 2D system defined by two dominant biological programs. The horizontal fibrotic–mechanical axis represents the degree of engagement in contractile and ECM-remodeling programs, ranging from homeostatic states (left) to highly fibrogenic, stiffness-driven states (right). The vertical inflammatory–stress axis represents the level of immune interaction, ranging from minimal immune engagement (bottom) to states explicitly driven by inflammatory cytokines (e.g., IL-1β, NF-κB) or interferon (IFN) signaling (top). Five stable attractor-like meta-states (A–E, large labeled circles) represent coarse-grained phenotype basins: **(A)** Homeostatic/Reservoir, **(B)** the central Myofibroblast Core, **(C)** Scar maintenance, **(D)** Immune-interactive, and **(E)** IFN-stressed. Associated marker genes, signaling pathways, niches, and specific fibroblast subtypes from the literature are noted in adjacent boxes. Half-filled circles indicate boundary states carrying mixed features between attractors (e.g., F-Trans/F-Act between **A** and **B**; Fibroblast-Clip between **B** and **C**). Solid lines suggest plausible trajectories between states, such as the progression from homeostasis **(A)** through repair **(B)** to chronic scarring **(C)**. Gray dashed arrows indicate the most suitable mapping attractor for the boundary state. Large colored arrows illustrate external microenvironmental inputs that exert forces on the system, biasing trajectories and stabilizing specific attractors: TGF-β and ECM stiffness feedback drives profibrotic differentiation toward scar maintenance (green arrow); chronic inflammatory cues from macrophages and T cells push the system toward the immune-interactive state (red arrow); and IFN niche signals drive the system toward the IFN-stressed phenotype (purple arrow).

To provide a comprehensive overview of these cell subtypes, we provide a qualitative conceptual framework that positions reported fibroblast subtype within a two-dimensional “force field” defined by a fibrotic–mechanical axis and an inflammatory–stress axis, and then uses this position to assign an attractor-like meta-state (A–E), boundary labels, and an interpretable trajectory role. Simultaneously, we provide a conceptual visualization to illustrate this framework (Figure 5).

The attractor-like meta-state here means a coarse-grained, stable phenotype basin. Many detailed fibroblast subtypes (with slightly different markers or niches) map into the same qualitative region of state-space because gene-regulatory and signaling interactions tend to produce preferred, self-stabilizing expression configurations (attractors). This idea comes from dynamical-systems views of cell fate, where cell states correspond to high-dimensional attractor states (Huang et al., 2005; Mojtahedi et al., 2016). In the 2D version, we do not claim to reconstruct the full high-dimensional landscape and instead we use two dominant “forces” (ECM/mechano drive and inflammatory stress) as a projection (Figure 5). A meta-state is attractor-like because it represents a region where trajectories would tend to flow in and persist unless strong external cues (e.g., ECM stiffening or IL-1β signaling inhibition) push the system across a boundary into another basin (Wang et al., 2011).

Operationally, the framework requires only qualitative annotations typically available in experimental reports—marker genes or proteins, named signaling pathways and transcriptional modules, spatial niche descriptions, cell–cell interaction narratives, and disease or model context—and converts them into intensity levels on each axis. For the x-axis, a low designation is appropriate when the description is dominated by quiescent or homeostatic matrix-maintenance features (e.g., stromal ECM housekeeping markers, no Acta2 and POSTN expression, and remote-zone context), implying minimal engagement of profibrotic differentiation programs (near the node A in Figure 5). A high designation is reserved for fibrogenic attractor-like states in which contractile and ECM programs are explicit (e.g., Acta2 high, collagen/POSTN-rich, and crosslinking/stiffness cues), or where the narrative emphasizes TGF-β–driven differentiation and the positive feedback between stiff ECM and mechanosignaling that stabilizes persistent activation (near the node C in Figure 5). For the y-axis, a low designation is used when immune interaction is not a defining feature (e.g., no macrophage/T-cell retention/IFN niche described, healthy/control or late resolved tissue without active inflammation). A high inflammatory designation (like node D or E in Figure 5) is warranted when the subtype is explicitly described as inflammatory or immune-interactive (e.g., IL-1/NF-κB/IL-6 signaling, explicit macrophage/T-cell wiring or IFN/ISG pathway).

Once a subtype is placed qualitatively in this 2D space, the framework assigns it to one of the meta-cluster attractors (A–E in Figure 5) that summarize recurrent fibroblast basins across studies while retaining interpretability. Attractor B (MYO) occupies a central position within the space, as it exhibits a dual nature (it is significant for wound closure and scar formation, yet it can lead to fibrosis when it persists or is aberrantly activated). Therefore, when allocating x-y coordinates to a novel subtype, MYO can be utilized as a reference. Another key conceptual point is that B and C are stabilized primarily by the x-axis feedback structure—TGF-β–driven profibrotic differentiation coupled to ECM stiffening and mechanotransduction—whereas D attractor are stabilized primarily by y-axis stress circuits and their niche reinforcement. In the presence of intense and persistent inflammatory stress, such as the interaction with CCR2^+^ macrophages, the subtype is more likely to be assigned to the region adjacent to the D attractor.

The 2D force-field framework is fast, data-light, and robust to the realities of cross-study comparison. It can be applied when only heterogeneous qualitative annotations are available (e.g., marker lists and niche notes), without requiring matched multi-omic measurements. Its core strength is interpretability—placing subtypes on a plane where “rightward” corresponds to stronger ECM/mechanical activation and “upward” corresponds to stronger inflammatory stress makes the mapping immediately legible to experimental and clinical audiences. Because the plane is explicitly designed to admit mixed drivers, it naturally accommodates boundary states—subtypes that carry both fibrotic and inflammatory features can be represented as intermediate regions rather than forced into mutually exclusive bins. Finally, the atlas functions as a practical front-end to a fuller systems pipeline. Once a subtype is positioned in 2D field, one can decide whether higher-resolution modeling (e.g., gene regulatory network module inference, spatial signaling graphs, or directed fate mapping with velocity-informed Markov transitions) is needed to resolve ambiguous cases (Lange et al., 2022).

The same design choices also create predictable limitations. The framework necessarily compresses multi-dimensional information, so important determinants of fibroblast heterogeneity that are partially orthogonal to both axes—such as lineage history, metabolic or senescent programs—may not be well separated in a 2D projection. Relatedly, each axis conflates multiple mechanisms. X-axis bundles synthesis, contractility, remodeling, and stiffness sensing, while y-axis combines many inflammatory pathways with type I IFN programs that can have distinct functional consequences. In addition, the atlas can narrate plausible trajectories (e.g., A→B→C), but it does not provide principled directionality, transition probabilities, or terminal-state likelihoods without bringing in external dynamical inference methods—an area where velocity- or time-informed Markov approaches are explicitly designed to estimate state-to-state transition structure (Weiler et al., 2024). Practical misclassification can also occur when studies lack key marker coverage (for example, missing IFN/ISG genes or stiffness/contractility markers), making axis placement underdetermined. Finally, while the 2D map suggests “which direction” an intervention should push the system (reduce x-drive or y-drive), it is less specific about which minimal set of drivers to perturb. By contrast, network control perspectives explicitly focus on how targeted control at selected nodes can steer complex multistable dynamics (MacLaren et al., 2025).

### 5.2 The limitations of single-cell transcriptomics in capturing spatial context

While scRNA-seq has been revolutionary for identifying the “what” (the different fibroblast subtypes), it does so at the cost of losing all spatial information by dissociating the tissue. By integrating scRNA-seq data with spatial transcriptomic maps, researchers can place each fibroblast subpopulation back into its native location (McLellan et al., 2020). This will allow for the identification of “pathological niches”, the specific microenvironments where pro-fibrotic fibroblasts interact with particular immune cells or stressed cardiomyocytes to drive disease (Cadosch et al., 2024). Understanding the cellular and molecular composition of these niches will be key to disrupting the pathogenic crosstalk that sustains fibrosis. A recent study identified a specific “fibrotic niche” in the ischemic zone of the heart (Chan et al., 2025). This niche is a hub of heterocellular communication involving macrophages, myofibroblasts, and T lymphocytes. The maturation of the cardiac scar is governed by a precise “mutual control” mechanism within this niche, initiating when lymphocytes secrete IL-4 to drive the differentiation of proinflammatory monocytes into a Trem2^+^ macrophage phenotype. These macrophages subsequently act to limit excessive fibrosis by secreting GAS6 (Growth Arrest-Specific 6) and PROS1 (Protein S), ligands that bind to the AXL receptor on fibroblasts to induce cell cycle arrest and senescence. Completing this reciprocal feedback loop, fibroblasts regulate the immune population by expressing Smad3, a ligand that binds to Nrp1 (Neuropilin-1) and Plxna (Plexin-A) receptors on the macrophages to suppress their proliferation. This coordinated “co-suppression” facilitates the maturation of the scar and prevents pathological fibrosis during late-stage cardiac repair (Chan et al., 2025).

### 5.3 The transcriptional to functional gap

While new techniques have provided a rich catalogue of novel fibroblast subtypes, the precise *in vivo* functions of many of these populations have yet to be validated. Identifying a subtype by biomarkers alone can lead to the “misidentification of therapeutic targets” because RNA expression does not always correlate with cellular behavior or protein-level function (Liu et al., 2016). A cell may express the mRNA for a receptor (biomarker), but never move that protein to the cell surface, making it an ineffective drug target. RNA biomarkers often capture a “snapshot” of a cell’s state (e.g., a stress response or a metabolic spike) rather than its stable lineage (Majima et al., 2024). Using these transient biomarkers to define a subtype can lead researchers to target a temporary state rather than the underlying disease-driving cell (Xu et al., 2024). Genetic gain- and loss-of-function studies using subtype-specific Cre-driver lines are needed to definitively establish their contributions to the fibrotic process (Shcholok and Eftekharpour, 2023).

### 5.4 The murine-to-human translation gap

Another primary challenge is the translation of these findings, predominantly from mouse models, to the more complex and heterogeneous human condition. Translating murine-identified biomarkers to human therapies is fraught with risk due to fundamental divergences in regulatory logic, environmental context, and expression patterns (Gros and Casanova, 2023). For instance, orthologous markers frequently exhibit distinct cellular distributions across species (Gilbertson and Weinmann, 2021). In murine models of sepsis and autoimmunity, scRNA-seq frequently identifies inflammatory monocyte subtypes via markers like Ly6C (Watanabe et al., 2024), leading to the therapeutic targeting of TLR4 to mitigate cytokine storms. While TLR4 is broadly expressed across various cell types in mice, its expression and signaling kinetics in humans are far more restricted to specific myeloid lineages (Stierschneider and Wiesner, 2023). This species-specific divergence explains why TLR4 antagonists like Eritoran showed immense promise in animal studies but ultimately failed in Phase III human trials (Opal et al., 2013), as the cellular “subtype” driving the pathology in mice did not functionally correspond to the target population in human patients (Chen et al., 2014). While key pathways and cell states like the FAP-MEOX1 axis appear to be conserved between mouse models and human heart failure (Alexanian et al., 2021), a much deeper characterization of fibroblast heterogeneity across the spectrum of human cardiac diseases is required.

To probe fibroblast heterogeneity and function in human condition, researchers have developed innovative models. Human induced pluripotent stem cell (hiPSC) technologies are now being used to model cardiac fibroblast development and disease. A study generated 3D self-organizing cardiac organoids containing cardiomyocytes, endothelial cells, and fibroblasts from hiPSCs (Song et al., 2024). By exposing these organoids to hypoxia and reoxygenation (to mimic ischemia-reperfusion injury), the model recapitulated key features of an acute MI, including cardiomyocyte death, contractile dysfunction, and notably fibrosis with collagen deposition. The fibroblasts within injured organoids activated and even exhibited myofibroblast-like behavior (increased α-SMA, ECM genes) and calcium handling changes similar to *in vivo* scar formation (Song et al., 2024). Another study focused on hPSC-derived epicardial cells, which were induced to undergo EMT and generate fibroblast-lineage cells *in vitro* (Fernandes et al., 2023). Strikingly, the resulting fibroblast populations in these organoid cultures showed a high degree of heterogeneity that mirrors adult fibroblast subtypes. The organoids contained fibroblast clusters analogous to those in a developing heart (fibroblasts and even adipocyte-like cells from epicardial lineage), as well as fibroblasts that transcriptionally matched those in adult human hearts. By simulating cardiac injury in these organoids, researchers identified a subpopulation of reparative fibroblasts (CD9^+^ fibroblasts) that closely resembles the pro-angiogenic, fibrosis-regulating fibroblasts observed in mouse injury models. This subset was characterized by markers like WIF1, DKK3, and CTHRC1, which are factors involved in antagonizing or fine-tuning Wnt signaling and collagen deposition. These CD9^+^ fibroblasts also expressed higher levels of genes such as SOX9, and COMP, matching the reparative fibroblast signature seen in scars (Fernandes et al., 2023). Such organoid platforms and lineage-specific differentiation systems are valuable for mechanistic testing. One can now culture fibroblast subtypes with cardiomyocytes to see how they differentially influence regeneration or scarring. They also offer means to screen drugs on specific fibroblast subpopulations (Fernandes et al., 2023).

Furthermore, are the fibroblast subtypes in HFpEF different from those in HFrEF (Li et al., 2021)? How do genetic cardiomyopathies alter the fibroblast landscape compared to hypertension-induced disease (Hall et al., 2021)? Answering these questions is crucial for tailoring therapies to specific patient populations. Finally, while the preclinical results of novel cell-based and antibody therapies are exciting, their long-term safety and efficacy in humans must be rigorously evaluated.

### 5.5 Future directions

Future work on fibroblast heterogeneity in cardiac fibrosis may concurrently progress in three complementary directions: (1) more precise genetic access to fibroblast states, (2) trajectory-resolved, multi-omic lineage tracing, and (3) *in situ* multi-omics to connect states to microanatomy and function. Because “fibroblast-specific” markers and tools remain imperfect, recent progress will depend on next-generation genetic models enabling precise labeling, tracing, and manipulation of fibroblast subsets. A representative example is intersectional dual recombinase fate mapping, where two orthogonal recombinases must both be active (e.g., a lineage driver plus a fibroblast driver), enabling high-specificity tracing and even selective ablation of a fibroblast subpopulation. In cardiac fibrosis, Han et al. used a dual inducible tracing strategy to identify and functionally test endocardium-derived fibroblasts as a pro-fibrotic subset (Han et al., 2023). In parallel, viral-genetic models are emerging because they can target subsets without germline engineering, for example, by coupling adeno-associated virus (AAV) tropism with state-selective promoters to drive effectors preferentially in activated cardiac fibroblasts after injury (Nakano et al., 2024). A further step is enhancer-driven AAV using injury-responsive tissue regeneration enhancer elements (TREEs) to achieve spatiotemporally restricted transgene expression in injured myocardium rather than healthy tissue (Wolfson et al., 2025). Multi-omic lineage barcoding approaches that capture clonal relationships across RNA and ATAC modalities provide a concrete blueprint for this kind of “state–fate” inference and can be adapted to fibroblast activation trajectories (Jindal et al., 2024). Finally, spatial multi-omics is becoming essential. Studies integrating single-cell and spatial transcriptomics in human heart demonstrate how fibroblast states map into discrete cellular niches and interaction circuits—an approach that can now be extended systematically across fibrotic etiologies to link fibroblast subtypes to regional ECM architecture, immune neighborhoods, and remodeling outcomes (Kanemaru et al., 2023).

Ultimately, these advances support a precision-medicine approach in which patients are stratified by fibrotic phenotype and treated with mechanism-matched interventions. For example, high FAP activity might motivate FAP-targeted strategies, whereas load-driven reactive fibrosis may be better addressed by targeting mechanosensing pathways (Ghazal et al., 2025). By embracing fibroblast heterogeneity, the field is positioned to move beyond one-size-fits-all therapy and develop more effective approaches to prevent, limit, and potentially reverse cardiac fibrosis.

## References

[B1] Aghajanian H. Kimura T. Rurik J. G. Hancock A. S. Leibowitz M. S. Li L. (2019). Targeting cardiac fibrosis with engineered T cells. Nature 573 (7774), 430–433. 10.1038/s41586-019-1546-z 31511695 PMC6752964

[B2] Aguado-Alvaro L. P. Garitano N. Pelacho B. (2024). Fibroblast diversity and epigenetic regulation in cardiac fibrosis. Int. J. Mol. Sci. 25 (11), 6004. 10.3390/ijms25116004 38892192 PMC11172550

[B3] Aguado-Alvaro L. P. Garitano N. Esser-Skala W. Sayers J. Del Valle C. Alameda D. (2025). Identification of epigenetic regulators of fibrotic transformation in cardiac fibroblasts through bulk and single-cell crispr screens. Nat. Commun. 16 (1), 11660. 10.1038/s41467-025-66597-9 41298482 PMC12749234

[B4] Alex L. Tuleta I. Hernandez S. C. Hanna A. Venugopal H. Astorkia M. (2023). Cardiac pericytes acquire a fibrogenic phenotype and contribute to vascular maturation after myocardial infarction. Circulation 148 (11), 882–898. 10.1161/CIRCULATIONAHA.123.064155 37350296 PMC10527624

[B5] Alexanian M. Padmanabhan A. Nishino T. Travers J. G. Ye L. Pelonero A. (2024). Chromatin remodelling drives immune cell-fibroblast communication in heart failure. Nature 635 (8038), 434–443. 10.1038/s41586-024-08085-6 39443808 PMC11698514

[B6] Alexanian M. Przytycki P. F. Micheletti R. Padmanabhan A. Ye L. Travers J. G. (2021). A transcriptional switch governs fibroblast activation in heart disease. Nature 595 (7867), 438–443. 10.1038/s41586-021-03674-1 34163071 PMC8341289

[B7] Amrute J. M. Luo X. Penna V. Yang S. Yamawaki T. Hayat S. (2024). Targeting immune-fibroblast cell communication in heart failure. Nature 635 (8038), 423–433. 10.1038/s41586-024-08008-5 39443792 PMC12334188

[B8] An D. Zeng Q. Zhang P. Ma Z. Zhang H. Liu Z. (2021). Alpha-ketoglutarate ameliorates pressure overload-induced chronic cardiac dysfunction in mice. Redox Biol. 46, 102088. 10.1016/j.redox.2021.102088 34364218 PMC8353361

[B9] Bachmann J. C. Baumgart S. J. Uryga A. K. Bosteen M. H. Borghetti G. Nyberg M. (2022). Fibrotic signaling in cardiac fibroblasts and vascular smooth muscle cells: the dual roles of fibrosis in hfpef and cad. Cells 11 (10), 1657. 10.3390/cells11101657 35626694 PMC9139546

[B10] Baudino T. A. Carver W. Giles W. Borg T. K. (2006). Cardiac fibroblasts: friend or foe? Am. J. Physiol. Heart Circ. Physiol. 291 (3), H1015–H1026. 10.1152/ajpheart.00023.2006 16617141

[B11] Bing R. Dweck M. R. (2019). Myocardial fibrosis: why image, how to image and clinical implications. Heart 105 (23), 1832–1840. 10.1136/heartjnl-2019-315560 31649047 PMC6900237

[B12] Bursac N. (2014). Cardiac fibroblasts in pressure overload hypertrophy: the enemy within? J. Clin. Invest 124 (7), 2850–2853. 10.1172/JCI76628 24937423 PMC4071394

[B13] Cadosch N. Gil-Cruz C. Perez-Shibayama C. Ludewig B. (2024). Cardiac fibroblastic niches in homeostasis and inflammation. Circ. Res. 134 (12), 1703–1717. 10.1161/CIRCRESAHA.124.323892 38843287 PMC11149942

[B14] Calcagno D. M. Taghdiri N. Ninh V. K. Mesfin J. M. Toomu A. Sehgal R. (2022). Single-cell and spatial transcriptomics of the infarcted heart define the dynamic onset of the border zone in response to mechanical destabilization. Nat. Cardiovasc Res. 1 (11), 1039–1055. 10.1038/s44161-022-00160-3 39086770 PMC11290420

[B15] Castoldi G. Carletti R. Ippolito S. Stella A. Zerbini G. Pelucchi S. (2021). Angiotensin type 2 and mas receptor activation prevents myocardial fibrosis and hypertrophy through the reduction of inflammatory cell infiltration and local sympathetic activity in angiotensin Ii-Dependent hypertension. Int. J. Mol. Sci. 22 (24), 13678. 10.3390/ijms222413678 34948475 PMC8708804

[B16] Chan A. S. Greiner J. Marschhauser L. Brennan T. A. Perez-Feliz S. Agrawal A. (2025). Spatiotemporal dynamics of the cardioimmune niche during lesion repair. Nat. Cardiovasc Res. 4 (11), 1550–1572. 10.1038/s44161-025-00739-6 41184578 PMC12611762

[B17] Chandekar K. R. Prashanth A. Vinjamuri S. Kumar R. (2023). Fapi Pet/Ct Imaging-an updated review. Diagn. (Basel) 13 (12), Epub 20230609. 10.3390/diagnostics13122018 37370912 PMC10297281

[B18] Chen P. Stanojcic M. Jeschke M. G. (2014). Differences between murine and human sepsis. Surg. Clin. North Am. 94 (6), 1135–1149. 10.1016/j.suc.2014.08.001 25440115 PMC7856627

[B19] Chen H. Hu K. Tang Q. Wang J. Gu Q. Chen J. (2025). Cd248-Targeted Bbir-T cell therapy against late-activated fibroblasts in cardiac repair after myocardial infarction. Nat. Commun. 16 (1), 2895. 10.1038/s41467-025-56703-2 40148319 PMC11950650

[B20] Chimenti I. Pagano F. Cozzolino C. Icolaro F. Floris E. Picchio V. (2025). The role of cardiac fibroblast heterogeneity in myocardial fibrosis and its novel therapeutic potential. Int. J. Mol. Sci. 26 (12), 5882. 10.3390/ijms26125882 40565343 PMC12193567

[B21] Chu C. Q. Quan T. (2024). Fibroblast yap/taz signaling in extracellular matrix homeostasis and tissue fibrosis. J. Clin. Med. 13 (12), 3358. 10.3390/jcm13123358 38929890 PMC11204269

[B22] Chung K. M. Hsu S. C. Chu Y. R. Lin M. Y. Jiaang W. T. Chen R. H. (2014). Fibroblast activation protein (fap) is essential for the migration of bone marrow mesenchymal stem cells through rhoa activation. PLoS One 9 (2), e88772. 10.1371/journal.pone.0088772 24551161 PMC3923824

[B23] Civitarese R. A. Talior-Volodarsky I. Desjardins J. F. Kabir G. Switzer J. Mitchell M. (2016). The Alpha11 integrin mediates fibroblast-extracellular matrix-cardiomyocyte interactions in health and disease. Am. J. Physiol. Heart Circ. Physiol. 311 (1), H96–H106. 10.1152/ajpheart.00918.2015 27199132

[B24] Creemers E. E. Pinto Y. M. (2011). Molecular mechanisms that control interstitial fibrosis in the pressure-overloaded heart. Cardiovasc Res. 89 (2), 265–272. 10.1093/cvr/cvq308 20880837

[B25] Cui Y. Wang Y. Wang S. Du B. Li X. Li Y. (2023). Highlighting fibroblasts activation in fibrosis: the state-of-the-art fibroblast activation protein inhibitor pet imaging in cardiovascular diseases. J. Clin. Med. 12 (18), 6033. 10.3390/jcm12186033 37762974 PMC10531835

[B26] Davis J. Molkentin J. D. (2014). Myofibroblasts: trust your heart and let fate decide. J. Mol. Cell Cardiol. 70, 9–18. 10.1016/j.yjmcc.2013.10.019 24189039 PMC3995855

[B27] Deng Y. He Y. Xu J. He H. Li G. (2023). Heterogeneity and functional analysis of cardiac fibroblasts in heart development. bioRxiv, 2023.07.30.551164. 10.1101/2023.07.30.551164 37577541 PMC10418062

[B28] Dewar M. B. Ehsan F. Izumi A. Zhang H. Zhou Y. Q. Shah H. (2024). Defining transcriptomic heterogeneity between left and right ventricle-derived cardiac fibroblasts. Cells 13 (4), 327. 10.3390/cells13040327 38391940 PMC10887120

[B29] Diez J. (2007). Mechanisms of cardiac fibrosis in hypertension. J. Clin. Hypertens. (Greenwich) 9 (7), 546–550. 10.1111/j.1524-6175.2007.06626.x 17617765 PMC8110048

[B30] Diez J. Gonzalez A. Kovacic J. C. (2020). Myocardial interstitial fibrosis in nonischemic heart disease, part 3/4: jacc focus seminar. J. Am. Coll. Cardiol. 75 (17), 2204–2218. 10.1016/j.jacc.2020.03.019 32354386 PMC7213023

[B31] Emig R. Zgierski-Johnston C. M. Beyersdorf F. Rylski B. Ravens U. Weber W. (2019). Human atrial fibroblast adaptation to heterogeneities in substrate stiffness. Front. Physiol. 10, 1526. 10.3389/fphys.2019.01526 31998137 PMC6965062

[B32] Fang N. Zhang N. Jiang X. Yan S. Wang Z. Gao Q. (2025). Pfkm-driven lactate overproduction promotes atrial fibrillation *via* triggering cardiac fibroblasts histone lactylation. Adv. Sci. (Weinh) 12 (34), e00963. 10.1002/advs.202500963 40569576 PMC12442653

[B33] Fernandes I. Funakoshi S. Hamidzada H. Epelman S. Keller G. (2023). Modeling cardiac fibroblast heterogeneity from human pluripotent stem cell-derived epicardial cells. Nat. Commun. 14 (1), 8183. 10.1038/s41467-023-43312-0 38081833 PMC10713677

[B34] Fiolet A. T. L. Opstal T. S. J. Mosterd A. Eikelboom J. W. Jolly S. S. Keech A. C. (2021). Efficacy and safety of low-dose colchicine in patients with coronary disease: a systematic review and meta-analysis of randomized trials. Eur. Heart J. 42 (28), 2765–2775. 10.1093/eurheartj/ehab115 33769515

[B35] Fitzgerald A. A. Weiner L. M. (2020). The role of fibroblast activation protein in health and malignancy. Cancer Metastasis Rev. 39 (3), 783–803. 10.1007/s10555-020-09909-3 32601975 PMC7487063

[B36] Floris E. Cozzolino C. Marconi S. Tonicello F. Picchio V. Pagano F. (2023). A review of therapeutic strategies against cardiac fibrosis: from classical pharmacology to novel molecular, epigenetic, and biotechnological approaches. Rev. Cardiovasc Med. 24 (8), 226. 10.31083/j.rcm2408226 39076707 PMC11266790

[B37] Forte E. Ramialison M. Nim H. T. Mara M. Li J. Y. Cohn R. (2022). Adult mouse fibroblasts retain organ-specific transcriptomic identity. Elife 11, 11. 10.7554/eLife.71008 35293863 PMC8959603

[B38] Frangogiannis N. G. (2014). The inflammatory response in myocardial injury, repair, and remodelling. Nat. Rev. Cardiol. 11 (5), 255–265. 10.1038/nrcardio.2014.28 24663091 PMC4407144

[B39] Frangogiannis N. G. (2019). The extracellular matrix in ischemic and nonischemic heart failure. Circ. Res. 125 (1), 117–146. 10.1161/CIRCRESAHA.119.311148 31219741 PMC6588179

[B40] Frangogiannis N. G. (2021). Cardiac fibrosis. Cardiovasc Res. 117 (6), 1450–1488. 10.1093/cvr/cvaa324 33135058 PMC8152700

[B41] Fu X. Khalil H. Kanisicak O. Boyer J. G. Vagnozzi R. J. Maliken B. D. (2018). Specialized fibroblast differentiated states underlie scar formation in the infarcted mouse heart. J. Clin. Invest 128 (5), 2127–2143. 10.1172/JCI98215 29664017 PMC5957472

[B42] Fuster-Martinez I. Calatayud S. (2024). The current landscape of antifibrotic therapy across different organs: a systematic approach. Pharmacol. Res. 205, 107245. 10.1016/j.phrs.2024.107245 38821150

[B43] Garoffolo G. Casaburo M. Amadeo F. Salvi M. Bernava G. Piacentini L. (2022). Reduction of cardiac fibrosis by interference with yap-dependent transactivation. Circ. Res. 131 (3), 239–257. 10.1161/CIRCRESAHA.121.319373 35770662

[B44] Garvin A. M. Khokhar B. S. Czubryt M. P. Hale T. M. (2021). Ras inhibition in resident fibroblast biology. Cell Signal 80, 109903. 10.1016/j.cellsig.2020.109903 33370581 PMC7878352

[B45] Gaudesius G. Miragoli M. Thomas S. P. Rohr S. (2003). Coupling of cardiac electrical activity over extended distances by fibroblasts of cardiac origin. Circ. Res. 93 (5), 421–428. 10.1161/01.RES.0000089258.40661.0C 12893743

[B46] Ghazal R. Wang M. Liu D. Tschumperlin D. J. Pereira N. L. (2025). Cardiac fibrosis in the multi-omics era: implications for heart failure. Circ. Res. 136 (7), 773–802. 10.1161/CIRCRESAHA.124.325402 40146800 PMC11949229

[B47] Gibb A. A. Lazaropoulos M. P. Elrod J. W. (2020). Myofibroblasts and fibrosis: mitochondrial and metabolic control of cellular differentiation. Circ. Res. 127 (3), 427–447. 10.1161/CIRCRESAHA.120.316958 32673537 PMC7982967

[B48] Gilbertson S. E. Weinmann A. S. (2021). Conservation and divergence in gene regulation between mouse and human immune cells deserves equal emphasis. Trends Immunol. 42 (12), 1077–1087. 10.1016/j.it.2021.10.007 34740529 PMC8616835

[B49] Gilles G. McCulloch A. D. Brakebusch C. H. Herum K. M. (2020). Maintaining resting cardiac fibroblasts *in vitro* by disrupting mechanotransduction. PLoS One 15 (10), e0241390. 10.1371/journal.pone.0241390 33104742 PMC7588109

[B50] Gros P. Casanova J. L. (2023). Reconciling mouse and human immunology at the altar of genetics. Annu. Rev. Immunol. 41, 39–71. 10.1146/annurev-immunol-101721-065201 36525691

[B51] Hall C. Gehmlich K. Denning C. Pavlovic D. (2021). Complex relationship between cardiac fibroblasts and cardiomyocytes in health and disease. J. Am. Heart Assoc. 10 (5), e019338. 10.1161/JAHA.120.019338 33586463 PMC8174279

[B52] Han M. Liu Z. Liu L. Huang X. Wang H. Pu W. (2023). Dual genetic tracing reveals a unique fibroblast subpopulation modulating cardiac fibrosis. Nat. Genet. 55 (4), 665–678. 10.1038/s41588-023-01337-7 36959363

[B53] Harrington A. Moore-Morris T. (2024). Cardiac fibroblasts in heart failure and regeneration. Front. Cell Dev. Biol. 12, 1388378. 10.3389/fcell.2024.1388378 38699159 PMC11063332

[B54] He X. Tolosa M. F. Zhang T. Goru S. K. Ulloa Severino L. Misra P. S. (2022). Myofibroblast yap/taz activation is a key step in organ fibrogenesis. JCI Insight 7 (4), e146243. 10.1172/jci.insight.146243 35191398 PMC8876427

[B55] Hinderer S. Schenke-Layland K. (2019). Cardiac fibrosis - a short review of causes and therapeutic strategies. Adv. Drug Deliv. Rev. 146, 77–82. 10.1016/j.addr.2019.05.011 31158407

[B56] Huang X. Hu L. Long Z. Wang X. Wu J. Cai J. (2024). Hypertensive heart disease: mechanisms, diagnosis and treatment. Rev. Cardiovasc Med. 25 (3), 93. 10.31083/j.rcm2503093 39076964 PMC11263885

[B57] Huang S. Eichler G. Bar-Yam Y. Ingber D. E. (2005). Cell fates as high-dimensional attractor states of a complex gene regulatory network. Phys. Rev. Lett. 94 (12), 128701. 10.1103/PhysRevLett.94.128701 15903968

[B58] Humeres C. Frangogiannis N. G. (2019). Fibroblasts in the infarcted, remodeling, and failing heart. JACC Basic Transl. Sci. 4 (3), 449–467. 10.1016/j.jacbts.2019.02.006 31312768 PMC6610002

[B59] Humeres C. Shinde A. V. Hanna A. Alex L. Hernandez S. C. Li R. (2022). Smad7 effects on Tgf-Beta and Erbb2 restrain myofibroblast activation and protect from postinfarction heart failure. J. Clin. Invest 132 (3), e146926. 10.1172/JCI146926 34905511 PMC8803336

[B60] Islam R. Hong Z. (2024). Yap/taz as mechanobiological signaling pathway in cardiovascular physiological regulation and pathogenesis. Mechanobiol. Med. 2 (4), 100085. 10.1016/j.mbm.2024.100085 39281415 PMC11391866

[B61] Ivey M. J. Tallquist M. D. (2016). Defining the cardiac fibroblast. Circ. J. 80 (11), 2269–2276. 10.1253/circj.CJ-16-1003 27746422 PMC5588900

[B62] Jindal K. Adil M. T. Yamaguchi N. Yang X. Wang H. C. Kamimoto K. (2024). Single-cell lineage capture across genomic modalities with Celltag-Multi reveals fate-specific gene regulatory changes. Nat. Biotechnol. 42 (6), 946–959. 10.1038/s41587-023-01931-4 37749269 PMC11180607

[B63] Kanemaru K. Cranley J. Muraro D. Miranda A. M. A. Ho S. Y. Wilbrey-Clark A. (2023). Spatially resolved multiomics of human cardiac niches. Nature 619 (7971), 801–810. 10.1038/s41586-023-06311-1 37438528 PMC10371870

[B64] Karur G. R. Aneja A. Stojanovska J. Hanneman K. Latchamsetty R. Kersting D. (2024). Imaging of cardiac fibrosis: an update, from the Ajr special series on imaging of fibrosis. AJR Am. J. Roentgenol. 222 (6), e2329870. 10.2214/AJR.23.29870 37753860

[B65] Kattih B. Boeckling F. Shumliakivska M. Tombor L. Rasper T. Schmitz K. (2023). Single-nuclear transcriptome profiling identifies persistent fibroblast activation in hypertrophic and failing human hearts of patients with longstanding disease. Cardiovasc Res. 119 (15), 2550–2562. 10.1093/cvr/cvad140 37648651

[B66] Khan R. Sheppard R. (2006). Fibrosis in heart disease: understanding the role of transforming growth factor-beta in cardiomyopathy, valvular disease and arrhythmia. Immunology 118 (1), 10–24. 10.1111/j.1365-2567.2006.02336.x 16630019 PMC1782267

[B67] King K. R. Aguirre A. D. Ye Y. X. Sun Y. Roh J. D. Ng R. P. Jr. (2017). Irf3 and type I interferons fuel a fatal response to myocardial infarction. Nat. Med. 23 (12), 1481–1487. 10.1038/nm.4428 29106401 PMC6477926

[B68] Kleinbongard P. Senyo S. E. Lindsey M. L. Garvin A. M. Simpson J. A. de Castro Braz L. E. (2024). Cardiac fibroblasts: answering the call. Am. J. Physiol. Heart Circ. Physiol. 327 (3), H681–H686. 10.1152/ajpheart.00478.2024 39093000 PMC11442096

[B69] Kong P. Christia P. Saxena A. Su Y. Frangogiannis N. G. (2013). Lack of specificity of fibroblast-specific protein 1 in cardiac remodeling and fibrosis. Am. J. Physiol. Heart Circ. Physiol. 305 (9), H1363–H1372. 10.1152/ajpheart.00395.2013 23997102 PMC3840245

[B70] Kong P. Christia P. Frangogiannis N. G. (2014). The pathogenesis of cardiac fibrosis. Cell Mol. Life Sci. 71 (4), 549–574. 10.1007/s00018-013-1349-6 23649149 PMC3769482

[B71] Lajiness J. D. Conway S. J. (2012). The dynamic role of cardiac fibroblasts in development and disease. J. Cardiovasc Transl. Res. 5 (6), 739–748. 10.1007/s12265-012-9394-3 22878976 PMC3740345

[B72] Lange M. Bergen V. Klein M. Setty M. Reuter B. Bakhti M. (2022). Cellrank for directed single-cell fate mapping. Nat. Methods 19 (2), 159–170. 10.1038/s41592-021-01346-6 35027767 PMC8828480

[B73] Learmonth M. Corker A. Dasgupta S. DeLeon-Pennell K. Y. (2023). Regulation of cardiac fibroblasts by lymphocytes after a myocardial infarction: playing in the major league. Am. J. Physiol. Heart Circ. Physiol. 325 (3), H553–H561. 10.1152/ajpheart.00250.2023 37450290 PMC10538980

[B74] Lendahl U. Muhl L. Betsholtz C. (2022). Identification, discrimination and heterogeneity of fibroblasts. Nat. Commun. 13 (1), 3409. 10.1038/s41467-022-30633-9 35701396 PMC9192344

[B75] Lewis G. A. Dodd S. Clayton D. Bedson E. Eccleson H. Schelbert E. B. (2021). Pirfenidone in heart failure with preserved ejection fraction: a randomized phase 2 trial. Nat. Med. 27 (8), 1477–1482. 10.1038/s41591-021-01452-0 34385704

[B76] Li P. Zhao H. Zhang J. Ning Y. Tu Y. Xu D. (2021). Similarities and differences between hfmref and hfpef. Front. Cardiovasc Med. 8, 678614. 10.3389/fcvm.2021.678614 34616777 PMC8488158

[B77] Li H. Zhu X. Cao X. Lu Y. Zhou J. Zhang X. (2023). Single-cell analysis reveals lysyl oxidase (Lox)(+) fibroblast subset involved in cardiac fibrosis of diabetic mice. J. Adv. Res. 54, 223–237. 10.1016/j.jare.2023.01.018 36706988 PMC10703720

[B78] Li G. Ni C. Wang J. Zhang F. Fu Z. Wang L. (2025). Dynamic molecular atlas of cardiac fibrosis at single-cell resolution shows Cd248 in cardiac fibroblasts orchestrates interactions with immune cells. Nat. Cardiovasc Res. 4 (4), 380–396. 10.1038/s44161-025-00617-1 40148545

[B79] Litvinukova M. Talavera-Lopez C. Maatz H. Reichart D. Worth C. L. Lindberg E. L. (2020). Cells of the adult human heart. Nature 588 (7838), 466–472. 10.1038/s41586-020-2797-4 32971526 PMC7681775

[B80] Liu Y. Beyer A. Aebersold R. (2016). On the dependency of cellular protein levels on Mrna abundance. Cell 165 (3), 535–550. 10.1016/j.cell.2016.03.014 27104977

[B81] Liu J. Feng J. Zhao J. Kong X. Yu Z. Huang Y. (2025). Tfap4 exacerbates pathological cardiac fibrosis by modulating mechanotransduction. Cell Insight 4 (4), 100256. 10.1016/j.cellin.2025.100256 40612272 PMC12226091

[B82] Luo C. Tan B. Chu L. Chen L. Zhong X. Jiang Y. (2024). Enhanced fibrotic potential of Col1a1(Hi)Nr4a1(Low) fibroblasts in ischemic heart revealed by transcriptional dynamics heterogeneity analysis at both bulk and single-cell levels. Front. Cardiovasc Med. 11, 1460813. 10.3389/fcvm.2024.1460813 39834736 PMC11743554

[B83] MacLaren N. G. Barzel B. Masuda N. (2025). Observing network dynamics through sentinel nodes. Nat. Commun. 16 (1), 10211. 10.1038/s41467-025-64975-x 41266296 PMC12635286

[B84] Majima K. Kojima Y. Minoura K. Abe K. Hirose H. Shimamura T. (2024). Lineagevae: reconstructing historical cell states and transcriptomes toward unobserved progenitors. Bioinformatics 40 (10), btae520. 10.1093/bioinformatics/btae520 39172488 PMC11494380

[B85] Maruyama K. Imanaka-Yoshida K. (2022). The pathogenesis of cardiac fibrosis: a review of recent progress. Int. J. Mol. Sci. 23 (5), 2617. 10.3390/ijms23052617 35269759 PMC8910720

[B86] McLellan M. A. Skelly D. A. Dona M. S. I. Squiers G. T. Farrugia G. E. Gaynor T. L. (2020). High-resolution transcriptomic profiling of the heart during chronic stress reveals cellular drivers of cardiac fibrosis and hypertrophy. Circulation 142 (15), 1448–1463. 10.1161/CIRCULATIONAHA.119.045115 32795101 PMC7547893

[B87] Mia M. M. Cibi D. M. Ghani S. Singh A. Tee N. Sivakumar V. (2022). Loss of Yap/Taz in cardiac fibroblasts attenuates adverse remodelling and improves cardiac function. Cardiovasc Res. 118 (7), 1785–1804. 10.1093/cvr/cvab205 34132780

[B88] Micheletti R. Alexanian M. (2022). Transcriptional plasticity of fibroblasts in heart disease. Biochem. Soc. Trans. 50 (5), 1247–1255. 10.1042/BST20210864 36281993 PMC9704531

[B89] Mojtahedi M. Skupin A. Zhou J. Castano I. G. Leong-Quong R. Y. Chang H. (2016). Cell fate decision as high-dimensional critical state transition. PLoS Biol. 14 (12), e2000640. 10.1371/journal.pbio.2000640 28027308 PMC5189937

[B90] Moore-Morris T. Guimaraes-Camboa N. Banerjee I. Zambon A. C. Kisseleva T. Velayoudon A. (2014). Resident fibroblast lineages mediate pressure overload-induced cardiac fibrosis. J. Clin. Invest 124 (7), 2921–2934. 10.1172/JCI74783 24937432 PMC4071409

[B91] Moore-Morris T. Guimaraes-Camboa N. Yutzey K. E. Puceat M. Evans S. M. (2015). Cardiac fibroblasts: from development to heart failure. J. Mol. Med. Berl. 93 (8), 823–830. 10.1007/s00109-015-1314-y 26169532 PMC4512919

[B92] Mouton A. J. Ma Y. Rivera Gonzalez O. J. Daseke M. J. 2nd Flynn E. R. Freeman T. C. (2019). Fibroblast polarization over the myocardial infarction time continuum shifts roles from inflammation to angiogenesis. Basic Res. Cardiol. 114 (2), 6. 10.1007/s00395-019-0715-4 30635789 PMC6329742

[B93] Nakano K. Sadahiro T. Fujita R. Isomi M. Abe Y. Yamada Y. (2024). Development of adeno-associated viral vectors targeting cardiac fibroblasts for efficient *in vivo* cardiac reprogramming. Stem Cell Rep. 19 (10), 1389–1398. 10.1016/j.stemcr.2024.08.002 39241770 PMC11561454

[B94] Nidorf S. M. Fiolet A. T. L. Mosterd A. Eikelboom J. W. Schut A. Opstal T. S. J. (2020). Colchicine in patients with chronic coronary disease. N. Engl. J. Med. 383 (19), 1838–1847. 10.1056/NEJMoa2021372 32865380

[B95] Ninh V. K. Calcagno D. M. Yu J. D. Zhang B. Taghdiri N. Sehgal R. (2024). Spatially clustered type I interferon responses at injury borderzones. Nature 633 (8028), 174–181. 10.1038/s41586-024-07806-1 39198639 PMC11374671

[B96] Niu L. Jia Y. Wu M. Liu H. Feng Y. Hu Y. (2020). Matrix stiffness controls cardiac fibroblast activation through regulating Yap Via at(1) R. J. Cell Physiol. 235 (11), 8345–8357. 10.1002/jcp.29678 32239716

[B97] Opal S. M. Laterre P. F. Francois B. LaRosa S. P. Angus D. C. Mira J. P. (2013). Effect of eritoran, an antagonist of Md2-Tlr4, on mortality in patients with severe sepsis: the access randomized trial. JAMA 309 (11), 1154–1162. 10.1001/jama.2013.2194 23512062

[B98] Parichatikanond W. Luangmonkong T. Mangmool S. Kurose H. (2020). Therapeutic targets for the treatment of cardiac fibrosis and cancer: focusing on Tgf-Beta signaling. Front. Cardiovasc Med. 7, 34. 10.3389/fcvm.2020.00034 32211422 PMC7075814

[B99] Park S. Nguyen N. B. Pezhouman A. Ardehali R. (2019). Cardiac fibrosis: potential therapeutic targets. Transl. Res. 209, 121–137. 10.1016/j.trsl.2019.03.001 30930180 PMC6545256

[B100] Patrick R. Janbandhu V. Tallapragada V. Tan S. S. M. McKinna E. E. Contreras O. (2024). Integration mapping of cardiac fibroblast single-cell transcriptomes elucidates cellular principles of fibrosis in diverse pathologies. Sci. Adv. 10 (25), eadk8501. 10.1126/sciadv.adk8501 38905342 PMC11192082

[B101] Peet C. Ivetic A. Bromage D. I. Shah A. M. (2020). Cardiac monocytes and macrophages after myocardial infarction. Cardiovasc Res. 116 (6), 1101–1112. 10.1093/cvr/cvz336 31841135 PMC7177720

[B102] Peisker F. Halder M. Nagai J. Ziegler S. Kaesler N. Hoeft K. (2022). Mapping the cardiac vascular niche in heart failure. Nat. Commun. 13 (1), 3027. 10.1038/s41467-022-30682-0 35641541 PMC9156759

[B103] Pinto A. R. Ilinykh A. Ivey M. J. Kuwabara J. T. D'Antoni M. L. Debuque R. (2016). Revisiting cardiac cellular composition. Circ. Res. 118 (3), 400–409. 10.1161/CIRCRESAHA.115.307778 26635390 PMC4744092

[B104] Porter K. E. Turner N. A. (2009). Cardiac fibroblasts: at the heart of myocardial remodeling. Pharmacol. Ther. 123 (2), 255–278. 10.1016/j.pharmthera.2009.05.002 19460403

[B105] Potere N. Bonaventura A. Abbate A. (2024). Novel therapeutics and upcoming clinical trials targeting inflammation in cardiovascular diseases. Arterioscler. Thromb. Vasc. Biol. 44 (12), 2371–2395. 10.1161/ATVBAHA.124.319980 39387118 PMC11602387

[B106] Prabhu S. D. Frangogiannis N. G. (2016). The biological basis for cardiac repair after myocardial infarction: from inflammation to fibrosis. Circ. Res. 119 (1), 91–112. 10.1161/CIRCRESAHA.116.303577 27340270 PMC4922528

[B107] Puhl S. L. Steffens S. (2019). Neutrophils in post-myocardial infarction inflammation: damage vs. Resolution? Front. Cardiovasc Med. 6, 25. 10.3389/fcvm.2019.00025 30937305 PMC6431642

[B108] Reichardt I. M. Robeson K. Z. Regnier M. Davis J. (2021). Controlling cardiac fibrosis through fibroblast state space modulation. Cell Signal 79, 109888. 10.1016/j.cellsig.2020.109888 33340659 PMC7856220

[B109] Ridker P. M. Everett B. M. Thuren T. MacFadyen J. G. Chang W. H. Ballantyne C. (2017). Antiinflammatory therapy with canakinumab for atherosclerotic disease. N. Engl. J. Med. 377 (12), 1119–1131. 10.1056/NEJMoa1707914 28845751

[B110] Ridker P. M. MacFadyen J. G. Thuren T. Libby P. (2020). Residual inflammatory risk associated with Interleukin-18 and Interleukin-6 after successful Interleukin-1beta inhibition with canakinumab: further rationale for the development of targeted anti-cytokine therapies for the treatment of atherothrombosis. Eur. Heart J. 41 (23), 2153–2163. 10.1093/eurheartj/ehz542 31504417

[B111] Romaine A. Sorensen I. W. Zeltz C. Lu N. Erusappan P. M. Melleby A. O. (2018). Overexpression of integrin Alpha11 induces cardiac fibrosis in mice. Acta Physiol. (Oxf) 222 (2). 10.1111/apha.12932 28771943

[B112] Roy J. Hettiarachchi S. U. Kaake M. Mukkamala R. Low P. S. (2020). Design and validation of fibroblast activation protein alpha targeted imaging and therapeutic agents. Theranostics 10 (13), 5778–5789. 10.7150/thno.41409 32483418 PMC7254991

[B113] Ruiz-Villalba A. Romero J. P. Hernandez S. C. Vilas-Zornoza A. Fortelny N. Castro-Labrador L. (2020). Single-cell rna sequencing analysis reveals a crucial role for Cthrc1 (collagen triple helix repeat containing 1) cardiac fibroblasts after myocardial infarction. Circulation 142 (19), 1831–1847. 10.1161/CIRCULATIONAHA.119.044557 32972203 PMC7730974

[B114] Rurik J. G. Tombacz I. Yadegari A. Mendez Fernandez P. O. Shewale S. V. Li L. (2022). Car T cells produced *in vivo* to treat cardiac injury. Science 375 (6576), 91–96. 10.1126/science.abm0594 34990237 PMC9983611

[B115] Sadaf S. Vasamsetti S. B. Jamal I. Johny E. Kubra K. T. Haque S. (2025). Activated fatty acid synthesis pathway in macrophages propagates pathogenic fibroblast expansion after myocardial infarction. bioRxiv, 2025.10.10.681697. 10.1101/2025.10.10.681697 41279812 PMC12632811

[B116] Schafer S. Viswanathan S. Widjaja A. A. Lim W. W. Moreno-Moral A. DeLaughter D. M. (2017). Il-11 is a crucial determinant of cardiovascular fibrosis. Nature 552 (7683), 110–115. 10.1038/nature24676 29160304 PMC5807082

[B117] Shcholok T. Eftekharpour E. (2023). Cre-recombinase systems for induction of neuron-specific knockout models: a guide for biomedical researchers. Neural Regen. Res. 18 (2), 273–279. 10.4103/1673-5374.346541 35900402 PMC9396489

[B118] Shi S. Y. Luo X. Yamawaki T. M. Li C. M. Ason B. Furtado M. B. (2021). Recent advances in single-cell profiling and multispecific therapeutics: paving the way for a new era of precision medicine targeting cardiac fibroblasts. Curr. Cardiol. Rep. 23 (7), 82. 10.1007/s11886-021-01517-z 34081224 PMC8175296

[B119] Shinde A. V. Frangogiannis N. G. (2014). Fibroblasts in myocardial infarction: a role in inflammation and repair. J. Mol. Cell Cardiol. 70, 74–82. 10.1016/j.yjmcc.2013.11.015 24321195 PMC3995820

[B120] Singh A. Bhatt K. S. Nguyen H. C. Frisbee J. C. Singh K. K. (2024). Endothelial-to-Mesenchymal transition in cardiovascular pathophysiology. Int. J. Mol. Sci. 25 (11), 6180. 10.3390/ijms25116180 38892367 PMC11173124

[B121] Snider P. Standley K. N. Wang J. Azhar M. Doetschman T. Conway S. J. (2009). Origin of cardiac fibroblasts and the role of periostin. Circ. Res. 105 (10), 934–947. 10.1161/CIRCRESAHA.109.201400 19893021 PMC2786053

[B122] Song M. Choi D. B. Im J. S. Song Y. N. Kim J. H. Lee H. (2024). Modeling acute myocardial infarction and cardiac fibrosis using human induced pluripotent stem cell-derived multi-cellular heart organoids. Cell Death Dis. 15 (5), 308. 10.1038/s41419-024-06703-9 38693114 PMC11063052

[B123] Stierschneider A. Wiesner C. (2023). Shedding light on the molecular and regulatory mechanisms of Tlr4 signaling in endothelial cells under physiological and inflamed conditions. Front. Immunol. 14, 1264889. 10.3389/fimmu.2023.1264889 38077393 PMC10704247

[B124] Stratton R. Shiwen X. (2010). Role of prostaglandins in fibroblast activation and fibrosis. J. Cell Commun. Signal 4 (2), 75–77. 10.1007/s12079-010-0089-8 20531982 PMC2876240

[B125] Tallquist M. D. Molkentin J. D. (2017). Redefining the identity of cardiac fibroblasts. Nat. Rev. Cardiol. 14 (8), 484–491. 10.1038/nrcardio.2017.57 28436487 PMC6329009

[B126] Talman V. Ruskoaho H. (2016). Cardiac fibrosis in myocardial infarction-from repair and remodeling to regeneration. Cell Tissue Res. 365 (3), 563–581. 10.1007/s00441-016-2431-9 27324127 PMC5010608

[B127] Tardif J. C. Kouz S. Waters D. D. Bertrand O. F. Diaz R. Maggioni A. P. (2019). Efficacy and safety of low-dose colchicine after myocardial infarction. N. Engl. J. Med. 381 (26), 2497–2505. 10.1056/NEJMoa1912388 31733140

[B128] Torimoto K. Elliott K. Nakayama Y. Yanagisawa H. Eguchi S. (2024). Cardiac and perivascular myofibroblasts, matrifibrocytes, and immune fibrocytes in hypertension; commonalities and differences with other cardiovascular diseases. Cardiovasc Res. 120 (6), 567–580. 10.1093/cvr/cvae044 38395029 PMC11485269

[B129] Travers J. G. Kamal F. A. Robbins J. Yutzey K. E. Blaxall B. C. (2016). Cardiac fibrosis: the fibroblast awakens. Circ. Res. 118 (6), 1021–1040. 10.1161/CIRCRESAHA.115.306565 26987915 PMC4800485

[B130] Trial J. Entman M. L. Cieslik K. A. (2016). Mesenchymal stem cell-derived inflammatory fibroblasts mediate interstitial fibrosis in the aging heart. J. Mol. Cell Cardiol. 91, 28–34. 10.1016/j.yjmcc.2015.12.017 26718722 PMC4764495

[B131] Tuleta I. Venugopal H. O'Leary K. Zheng D. Frangogiannis N. G. (2025). Fibroblast-specific loss of Tgf-Beta signaling mediates lipomatous metaplasia in the infarcted heart. Circulation 152 (20), 1423–1435. 10.1161/CIRCULATIONAHA.125.075676 40970279 PMC12576608

[B132] Umbarkar P. Ejantkar S. Tousif S. Lal H. (2021). Mechanisms of fibroblast activation and myocardial fibrosis: lessons learned from Fb-Specific conditional mouse models. Cells 10 (9), 2412. 10.3390/cells10092412 34572061 PMC8471002

[B133] Venugopal H. Hanna A. Humeres C. Frangogiannis N. G. (2022). Properties and functions of fibroblasts and myofibroblasts in myocardial infarction. Cells 11 (9), 1386. 10.3390/cells11091386 35563692 PMC9102016

[B134] Wang J. Zhang K. Xu L. Wang E. (2011). Quantifying the waddington landscape and biological paths for development and differentiation. Proc. Natl. Acad. Sci. U. S. A. 108 (20), 8257–8262. 10.1073/pnas.1017017108 21536909 PMC3100956

[B135] Wang Y. Li Q. Tao B. Angelini M. Ramadoss S. Sun B. (2023). Fibroblasts in heart scar tissue directly regulate cardiac excitability and arrhythmogenesis. Science 381 (6665), 1480–1487. 10.1126/science.adh9925 37769108 PMC10768850

[B136] Wang H. Yang J. Cai Y. Zhao Y. (2024). Macrophages suppress cardiac reprogramming of fibroblasts *in vivo* Via Ifn-Mediated intercellular self-stimulating circuit. Protein Cell 15 (12), 906–929. 10.1093/procel/pwae013 38530808 PMC11637486

[B137] Wang Z. Zhang Z. Ping Z. Yang S. Li Y. Jiang T. (2025). Succinate-driven Pkm2 succinylation and dimerization accelerates age-associated cardiac fibrosis. Commun. Biol. 10.1038/s42003-025-09337-5 41372616 PMC13168700

[B138] Watanabe H. Rana M. Son M. Chiu P. Y. Fei-Bloom Y. Choi K. (2024). Single cell rna-seq reveals cellular and transcriptional heterogeneity in the splenic Cd11b(+)Ly6c(High) monocyte population expanded in sepsis-surviving mice. Mol. Med. 30 (1), 202. 10.1186/s10020-024-00970-0 39506629 PMC11539566

[B139] Weiler P. Lange M. Klein M. Pe'er D. Theis F. (2024). Cellrank 2: unified fate mapping in multiview single-cell data. Nat. Methods 21 (7), 1196–1205. 10.1038/s41592-024-02303-9 38871986 PMC11239496

[B140] Wolfson D. W. Hull J. A. Li Y. Gonzalez T. J. Jayaram M. D. Devlin G. W. (2025). Spatial and longitudinal tracking of enhancer-aav vectors that target transgene expression to injured mouse myocardium. bioRxiv, 2025.04.28.651096. 10.1101/2025.04.28.651096 40938325 PMC12431772

[B141] Xu X. Wen Q. Lan T. Zeng L. Zeng Y. Lin S. (2024). Time-resolved single-cell transcriptomic sequencing. Chem. Sci. 15 (46), 19225–19246. 10.1039/d4sc05700g 39568874 PMC11575584

[B142] Yucel D. Ferreira de Araujo N. Souza-Neto F. Smith C. Lin W. H. Torniainen A. A. (2025). Septin4 regulates cardiac fibrosis after pressure overload. Circ. Res. 137 (8), 1117–1132. 10.1161/CIRCRESAHA.125.326758 40960950 PMC12466173

[B143] Zeisberg E. M. Kalluri R. (2010). Origins of cardiac fibroblasts. Circ. Res. 107 (11), 1304–1312. 10.1161/CIRCRESAHA.110.231910 21106947 PMC3098499

